# CD1^−^ and CD1^+^ porcine blood dendritic cells are enriched for the orthologues of the two major mammalian conventional subsets

**DOI:** 10.1038/srep40942

**Published:** 2017-01-20

**Authors:** Jane C. Edwards, Helen E. Everett, Miriam Pedrera, Helen Mokhtar, Emanuele Marchi, Ferran Soldevila, Daryan A. Kaveh, Philip J. Hogarth, Helen L. Johns, Javier Nunez-Garcia, Falko Steinbach, Helen R. Crooke, Simon P. Graham

**Affiliations:** 1 Virology Department, Animal and Plant Health Agency, Addlestone, KT15 3NB, UK; 2 Nuffield Department of Medicine, University of Oxford, Oxford, OX1 3SY, UK; 3 Flow Cytometry Facility, Animal and Plant Health Agency, Addlestone, KT15 3NB, UK; 4 Bacteriology Department, Animal and Plant Health Agency, Addlestone, KT15 3NB, UK; 5 Epidemiology Department, Animal and Plant Health Agency, Addlestone, KT15 3NB, UK

## Abstract

Conventional dendritic cells (cDC) are professional antigen-presenting cells that induce immune activation or tolerance. Two functionally specialised populations, termed cDC1 and cDC2, have been described in humans, mice, ruminants and recently in pigs. Pigs are an important biomedical model species and a key source of animal protein; therefore further understanding of their immune system will help underpin the development of disease prevention strategies. To characterise cDC populations in porcine blood, DC were enriched from PBMC by CD14 depletion and CD172a enrichment then stained with lineage mAbs (Lin; CD3, CD8α, CD14 and CD21) and mAbs specific for CD172a, CD1 and CD4. Two distinct porcine cDC subpopulations were FACSorted CD1^−^ cDC (Lin^−^CD172^+^ CD1^−^CD4^−^) and CD1^+^ cDC (Lin^−^CD172a^+^ CD1^+^ CD4^−^), and characterised by phenotypic and functional analyses. CD1^+^ cDC were distinct from CD1^−^ cDC, expressing higher levels of CD172a, MHC class II and CD11b. Following TLR stimulation, CD1^+^ cDC produced IL-8 and IL-10 while CD1^−^ cDC secreted IFN-α, IL-12 and TNF-α. CD1^−^ cDC were superior in stimulating allogeneic T cell responses and in cross-presenting viral antigens to CD8 T cells. Comparison of transcriptional profiles further suggested that the CD1^−^ and CD1^+^ populations were enriched for the orthologues of cDC1 and cDC2 subsets respectively.

Dendritic cells (DC) were first identified in the peripheral lymphoid organs of mice^1^ and are regarded as the sentinels of the immune system. Often resident in tissues close to sites of pathogen entry, DC take up antigen and migrate to lymphoid organs where they present antigen to T cells^2^. DC are unique in their capacity to activate naïve T cells^3^ but also play a pivotal role in maintaining central tolerance to self-antigen^4^. DC can be broadly classified into two lineage populations; plasmacytoid DC (pDC), specialising in production of cytokines, most notably type I IFNs^5^, and conventional DC (cDC), which are potent antigen-presenting cells (APCs)^6^. In the mouse, splenic cDC populations were further delineated based on expression of CD8α and CD11b (CD8α^+^ CD11b^−^ and CD8α^−^CD11b^+^)^7^. CD8α^+^ cDC express XCR1, TLR3^8^, produce IL-12^9^,^10^ and are highly efficient at cross-presenting exogenous antigen to CD8^+^ T cells^11^,^12^,^13^. They are specialised in the uptake of apoptotic bodies^13^ and are generally located in the T cell areas of the Peyer’s patches and the spleen^14^. Mice lacking XCR1 or its ligand, are less able to cross-present antigen necessary for induction of CD8^+^ T cell responses against various viruses and bacteria^7^,^15^. In contrast, the CD11b^+^ subset of cDC are located in areas associated with antigen uptake, including the marginal zone and sub-epithelial dome of secondary lymphoid tissues, and show high rates of endocytosis and phagocytosis^16^. CD11b^+^ DC also express high levels of proteins involved in MHC class II presentation and are most efficient at inducing CD4^+^ Th2 responses, whereas Th1 responses are preferentially induced by CD8α^+^ cDC^9^,^17^,^18^.

The CD8α^+^ CD11b^−^ and CD8α^−^CD11b^+^ populations have now been classified as cDC1 and cDC2 respectively with a conserved phenotype and function seen across several mammalian species^19^. For example, the human CD141^+^ cDC subset in blood is equivalent to the mouse cDC1, sharing expression of CLEC9a^20^,^21^,^22^, XCR1^22^,^23^, CADM1, TLR3, BAFT3 and IRF8^24^,^25^. These cells also produce type III IFN^26^ following activation with a TLR3 agonist. However, unlike the mouse the unique capacity for effective cross-presentation by the human cDC1 subset is more controversial^27^,^28^; while some studies have demonstrated that cDC1 DCs are superior^22^,^23^,^29^, others have concluded that tonsillar cDC1 possess a comparable capacity to cDC2^30^. Others have shown that TLR3 stimulation is necessary for blood-derived cDC1 to efficiently cross-present, but this was not required for skin derived cDC1^31^. Certainly the precise conditions, such as the source of cDC and the nature of the antigen, are likely to play a role in influencing cross-presentation, in humans and possibly other mammalian species. In comparison, human CD1c^+^ cDC2 express higher levels of mRNA associated with MHC class II antigen processing including up-regulation of cathepsin H^29^. A comparative analysis of the transcriptomes of human and murine cDC subsets has shown marked similarity between murine splenic CD11b^+^ and CD8α^+^ cDC and human blood CD1c^+^ and CD141^+^ cDC, respectively^24^,^32^. Transcriptional and functional profiling has further demonstrated that the two major cDC populations are also conserved in sheep^33^ and macaques^34^. Ovine efferent lymph CD26^+^ CD172a^−^ cDC share properties with cDC1, including expression of transcription factors ID2, IRF8, BATF3, the membrane proteins CLEC9a and CADM1, IL-12, and were superior to CD26^−^CD172a^+^ cDC in their ability to activate antigen-specific CD8 T cells^33^.

The pig represents an economically significant livestock species and an important large animal model for biomedical research in fields such as xenotransplantation and influenza infection biology. With the intention of identifying cDC in the skin as targets for vaccination strategies others have demonstrated that porcine skin CD163^low^ cells share phenotypic and transcriptomic features consistent with the cDC2, and a CD172a^−^ subset orthologous to cDC1 cells^35^,^36^. Similar populations have also recently been identified in the porcine lung^37^. In addition to providing new avenues for DC-targeted vaccine approaches, definition of the phenotype and function of cDC subsets in the pig will enable an improved understanding of the interaction of these cells with pathogens, including a number of globally economically important myelotropic viruses such as classical swine fever, African swine fever, and porcine reproductive and respiratory syndrome viruses. Blood represents an easily accessible tissue, enabling repeated sampling from live animals which supports the reduction in use of animals in scientific research. In porcine blood, cDC have been identified as possessing the lineage^−^CD172a^+^ phenotype^38^,^39^,^40^ which are further delineated on the basis of CD4 expression into CD4^+^ pDC and CD4^−^ cDC^38^. Given the expression of CD1 and CD11b on a subpopulation of porcine cDC^38^,^39^, we hypothesised that this subset may be analogous to cDC2 and conversely that porcine CD1^−^CD11b^−^ cDC might be enriched for the equivalent to cDC1. Through a combination of phenotypic and functional analysis as well as comparative transcriptomics we show here that the porcine blood CD1^+^ cDC population is orthologous to cDC2 cells described in other mammals. The CD1^−^ cDC subset, contains both XCR1^hi^ cells previously described in other mammals as cDC1 cells, but also cDC expressing varying levels of CADM1 and XCR1 potentially representing variants of cDC1 DC at different stages of maturation or activation.

## Results

### Sorting and phenotypic characterisation of porcine blood dendritic cells

**Figure 1:**
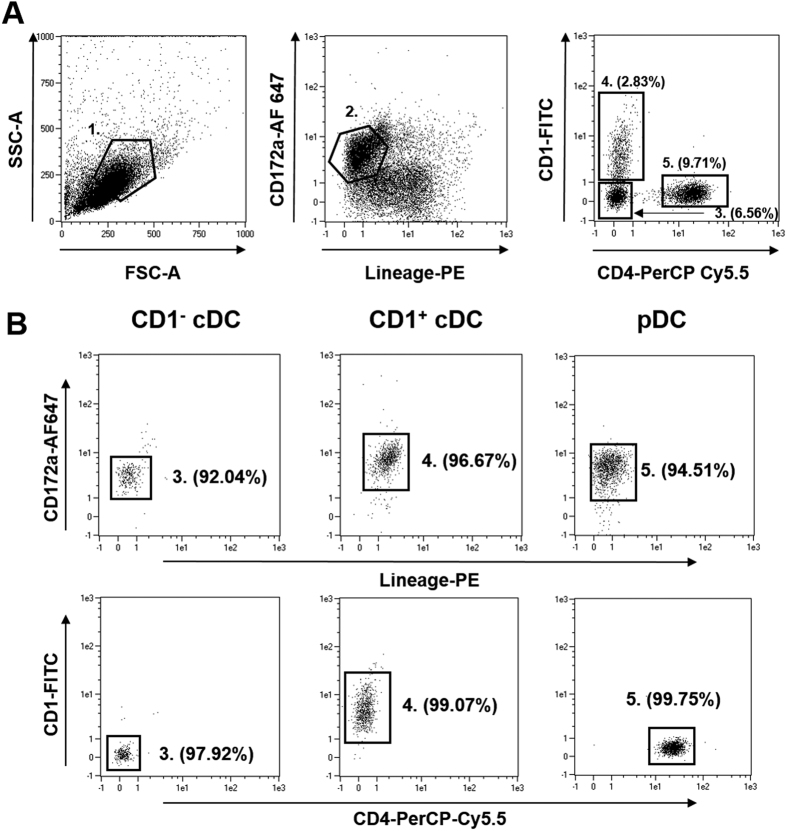
Sorting strategy for the isolation of porcine blood DC populations. Blood DC, enriched by magnetic depletion of CD14^+^ cells and selection of CD172a^+^ cells, were stained with mAbs to CD172a and lineage markers (lin; CD3, CD8α, CD21) (**A**). Large (gate 1) CD172a^+^ lin^−^ (gate 2) blood DC subsets were then sorted on expression CD1 and CD4: CD4^−^CD1^−^ cDC (gate 3), CD4^−^CD1^+^ cDC (gate 4) and CD1^−^CD4^+^ pDC (gate 5). Sorted blood DC subsets showed >95% purity when assessed by flow cytometry (**B**).

CD172a (SIRP-α) is expressed on porcine DC circulating in blood^38^,^40^. Monocytes circulate at significantly higher numbers than DC in the blood and also express CD172a albeit at a higher level than observed on the surface of DC^41^. To permit the successful enrichment of highly pure populations of blood DC, PBMC were first depleted of monocytes using the monocyte specific marker CD14^42^,^43^ and the resulting CD14 negative fraction enriched for CD172a^+^ cells using magnetic-based cell sorting (Fig. 1A). The resulting CD14^−^CD172a^+^ enriched DC population was stained with fluorochrome-labelled antibodies to CD172a, lineage markers (CD3, CD8α, CD14, CD21), CD1 and CD4 prior to flow cytometric cell sorting (Fig. 1B). Blood DC were firstly identified as CD172a^+^ lin^−^ cells, and CD172a^high^ cells were excluded since it has been shown that Tuk4 antibody for CD14 may not identify all monocytes^44^,^45^. Two populations of CD172a^+^ lin^−^ cells could be identified; CD4^−^CD1^−^ and CD4^−^CD1^+^; while pDC were identified as CD4^+^ CD1^−^ cells (Fig. 1A) as previously reported^38^. After sorting, the purity of the three populations was assessed both for contamination by lymphocytes and other DC subsets by flow cytometric analysis and were typically >95% pure (Fig. 1B). Notably, we observed that the cDC express different levels of CD172a (Fig. 1B). Staining these populations directly in intact PBMC (i.e. non-CD14 depleted and non-CD172a enriched PBMC) showed that these cells circulate at approximately 0.5–1% (pDC), 0.1% (CD1^+^ cDC) and 0.1% (CD1^−^ cDC) of the total PBMC population (Supplementary Figure S1).

**Figure 2:**
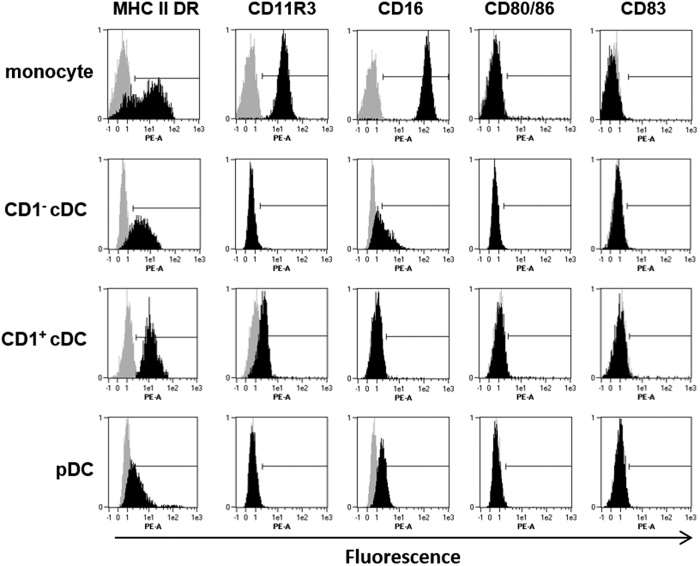
Sorted porcine blood DC populations and monocytes express distinct cell surface phenotypes. Freshly isolated monocytes, CD1^−^ cDC, CD1^+^ cDC and pDC were stained with a panel of DC markers (black histograms) and corresponding host/isotype matched control antibodies (grey histograms) and analysed by flow cytometry. Representative data is shown from 1 of 3 individual pigs analysed.

To further delineate the 3 populations of freshly sorted blood DC, cells were stained with a panel of antibodies directed to markers known to be expressed on porcine monocyte-derived DC^46^ and DC in human blood^47^ (Fig. 2). Purified monocytes were included for comparison. Since the individual cell populations varied in both their levels of autofluorescence and spill-over fluorescence associated with the specific antibodies employed for sorting, staining with isotype control antibodies was used to normalise the results across the four cell populations and confine the negative controls to the first log decade. MHC class II (MHC-II) expression by monocytes showed a biphasic profile, most likely representing mature and immature populations of monocytes previously described in pig with differing levels of MHC-II^42^. In contrast, each blood DC population showed relatively uniform expression of MHC-II suggesting isolation of homogenous populations. Freshly isolated pDC expressed very low levels of MHC-II as has been previously described^38^. CD11R3, believed to be the orthologue of human CD11b, was expressed at high levels on monocytes, as shown previously in pigs^43^ and humans (CD11b)^48^ and also on CD1^+^ cDC. There was no expression on CD1^−^ cDC and pDC. Differential levels of CD16 expression were also observed; CD1^+^ cDC lacked CD16 expression and pDC expressed very little, while slightly higher levels were observed on some CD1^−^ cDC. All freshly isolated populations lacked expression of CD83 and the co-stimulatory complex CD80/86, as assessed by staining with a CTLA-4 fusion protein.

**Figure 3:**
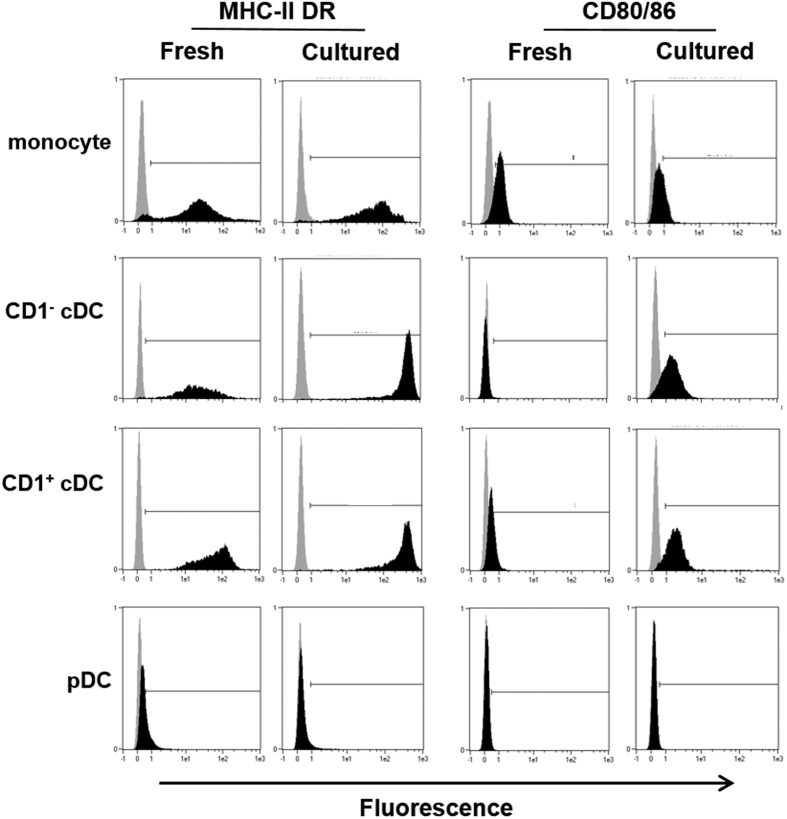
Assessment of the effect of cell culture on the phenotype of sorted porcine blood DC populations and monocytes. Expression of MHC class II DR and CD80/86 by monocytes, CD1^−^ cDC, CD1^+^ cDC and pDC upon isolation (fresh) or following an 18 h culture (cultured) was assessed by flow cytometry. MHC class II DR and CD80/86 staining (black histograms) was compared against the corresponding isotype control antibody (grey histograms) and representative data is shown from 1 of 3 individual pigs analysed.

To determine if the isolated cDC populations gained an antigen presenting phenotype upon culture, DC were stained for MHC-II DR and CD80/86 following 18 h culture in the presence of IL-3 at 37 °C, 5% CO_2_ (Fig. 3). Both CD1^−^ and CD1^+^ cDC showed extremely high levels of MHC-II expression, and gained significant expression of CD80/86 on their surface, upon culture. Culture of monocytes induced only a modest up-regulation of MHC-II expression while both markers remained unchanged on cultured pDC.

### Cytokine responses to stimulation with pathogen-associated molecular patterns (PAMPs)

**Figure 4:**
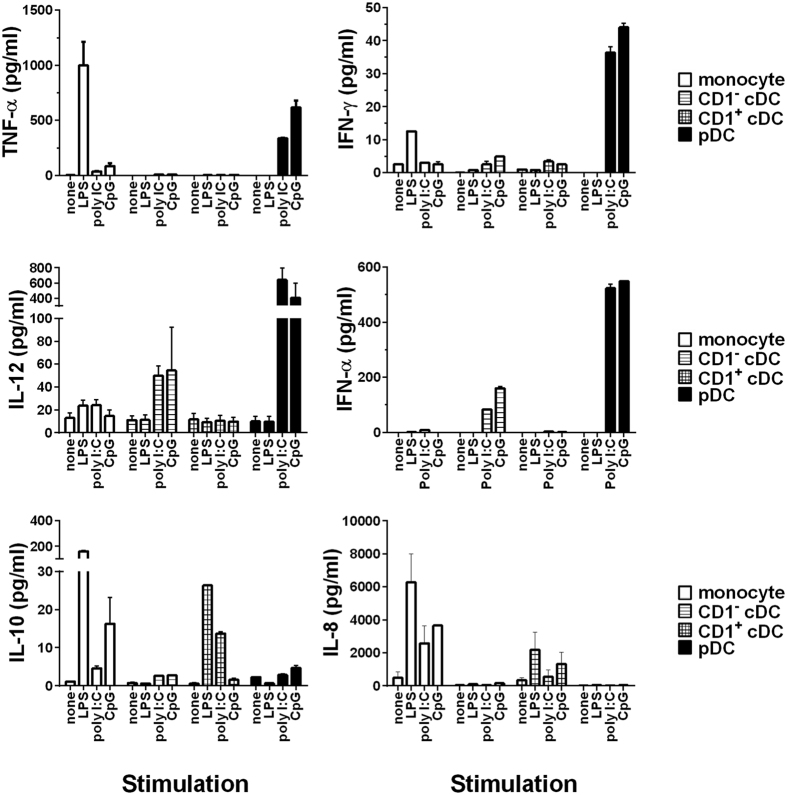
Differential responses of sorted porcine blood DC populations and monocytes to PAMP stimulation. Monocytes, CD1^−^ cDC, CD1^+^ cDC and pDC were cultured in the presence of poly(I:C), LPS, CpG-ODN 2007 or in medium alone for 18 h. Cytokine content of cell-free culture supernatants were then assessed by multiplex (TNF-α, IFN-γ, IFN-α, IL-10 and IL-8) or singleplex (IL-12) ELISAs and data presented as the mean cytokine concentration of triplicate pooled supernatants from 3 pigs ± SEM.

DC express a broad repertoire of toll-like receptors (TLR) which recognise pathogen-associated molecular patterns (PAMPs) which serve to activate the DC, resulting in maturation and release of cytokines which polarise T cell differentiation^49^. However, expression of TLRs is not uniform across DC subsets^8^, suggesting functional specificity towards various pathogens^8^. For instance, TLR3 is expressed at the highest levels on CD8α^+^ cDC in mice^8^ and CD141^+^ cDC in humans^29^,^50^ while TLR4 expression is restricted to BDCA-1^+^ cDC^29^. To assess responses to PAMP stimulation amongst the porcine DC populations, we stimulated sorted blood DC with three prototypic PAMPs; LPS (recognised by TLR4), poly (I:C) (recognised by TLR3 and also by cytosolic RNA helicases retinoic acid-inducible protein I (RIG-I) and melanoma differentiation-associate gene 5 (MDA-5)) and Class B CpG-ODN 2007 (recognised by TLR9) and compared their cytokine secretion profiles across the three pig DC populations (Fig. 4). CD1^−^ cDC responded to poly(I:C) and CpG-ODN, but not LPS, with secretion of IL-12, IFN-γ and IFN-α, although only the data for IFN-γ (CpG-ODN, p < 0.05) and IFN-α (poly I:C p < 0.0001, CpG-ODN p < 0.0001) were statistically significant compared with unstimulated cells (probably caused by the inter-animal variability in the cytokine levels). In contrast, CD1^+^ cDC responded to LPS (p < 0.0001) and poly(I:C) (p < 0.01) with secretion of IL-10, and to LPS and CpG-ODN with IL-8. The pDC responded exclusively to poly(I:C) and CpG-ODN, with high levels of TNF-α (poly I:C, p < 0.01, CpG-ODN, p < 0.0001) IL-12 (poly I:C, p < 0.0001, CpG-ODN, p < 0.001), IFN-α (poly I:C, p < 0.0001, CpG-ODN, p < 0.0001), and IFN-γ (poly I:C, p < 0.0001, CpG-ODN, p < 0.0001). In comparison, monocytes responded to LPS with high levels of TNFα (p < 0.0001) and IFN-γ (p < 0.001). They also secreted IL-10 and IL-8 in response to LPS (p < 0.001) and CpG (p < 0.01).

### Assessment of antigen uptake, processing and presentation by porcine blood DCs

**Figure 5:**
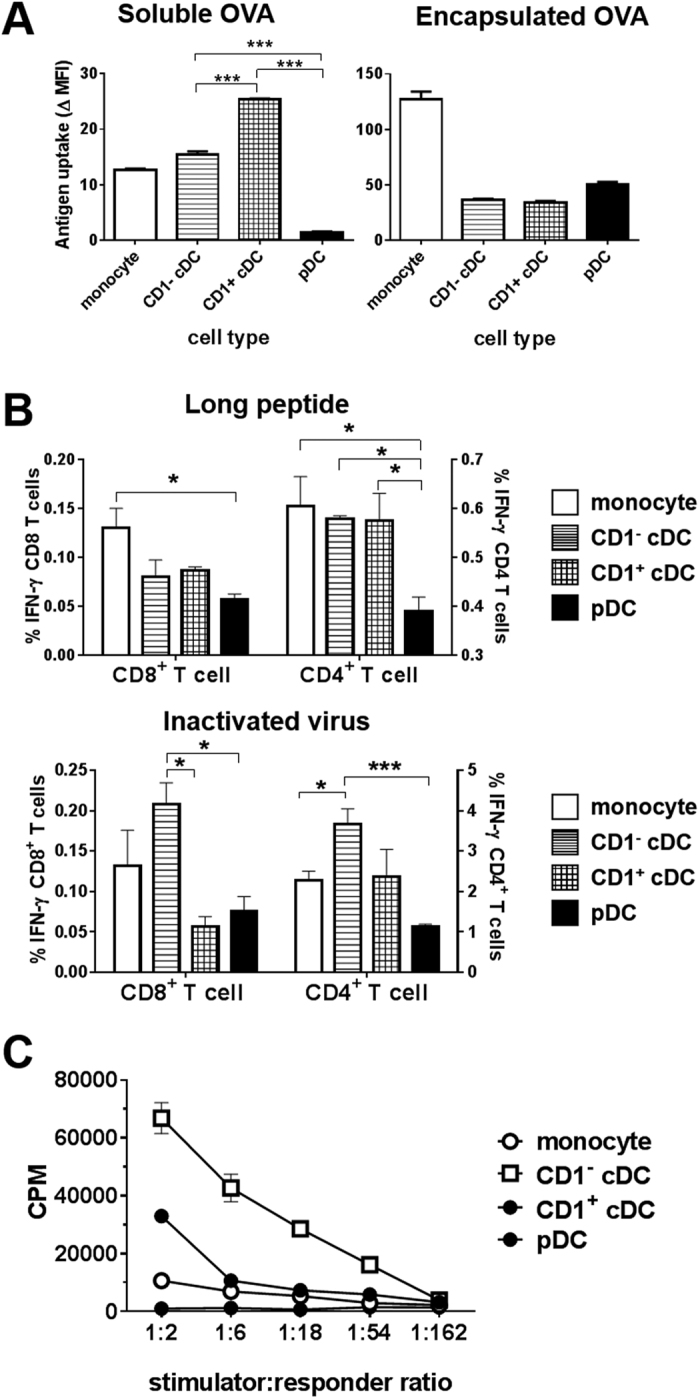
Assessment of the antigen uptake, processing and presentation capabilities of porcine blood DC populations and monocytes. The ability of blood DC populations and monocytes to endocytose soluble and phagocytose particulate antigen was examined using Alexa Fluor-647^®^-conjugated ovalbumin either in soluble form or encapsulated in PLGA nanoparticles. Antigen uptake was determined after 1 h by flow cytometry. Antigen uptake was measured by mean fluorescence intensity measurements and data presented are the mean 4 °C corrected antigen uptake at 37 °C for triplicate cultures from 1/3 representative experiments (**A**). Stimulation of antigen-specific CD4^+^ and CD8^+^ T cell IFN-γ responses by sorted blood DC populations and monocytes pulsed with a 28mer synthetic peptide carrying defined CD4 and CD8 T cell epitopes (long peptide) or inactivated PRRSV was assessed by flow cytometry. The unstimulated or, in the case of PRRSV, the mock-virus stimulated corrected mean % IFN-γ^+^ live, singlet CD8 (CD4^−^CD8α^high^; left y-axis) and memory CD4 (CD4^+^ CD8α^low^; right y-axis) T cells for triplicate cultures from 1 of 2 experiments are presented (**B**). Allogeneic T cell stimulatory capacity of monocytes and DC populations was assessed in a mixed-leukocyte reaction and lymphoproliferation assessed by ^3^H-thymidine incorporation. The data are presented as the mean incorporated counts per minute (cpm) of triplicate cultures ± SEM from 1 of 2 experiments (**C**). For all plots error bars represent SEM. Values were compared using a two-tailed un-paired t-test and significance indicated by ***p < 0.001, **p < 0.01, *p < 0.05.

To gain further insight into the functional specialisation of the isolated DC populations, we compared the capacity of the cells to take up antigen, delivered either as soluble Alexa Fluor-647^®^-conjugated ovalbumin (OVA-AF647) or OVA-AF647 encapsulated in PLGA nanoparticles (Fig. 5A). Both cDC populations took up greater amounts of soluble OVA-AF647 levels compared with pDC (p < 0.001). However, CD1^+^ cDC were superior in their ability to take up soluble OVA-AF647 compared with CD1^−^ cDC (p < 0.001). In contrast, there was no difference in the ability of DC populations to take up particulate antigen, which was most efficiently endocytosed by monocytes.

Murine CD8^+^ cDC,^13^,^51^ ovine CD26^+^ cDC^33^ and human CD141^+^ cDC^21^,^23^,^29^ all have a superior capacity for cross-presenting exogenous antigen to CD8 T cells, which probably reflects their specialisation in the induction of immunity against intracellular pathogens. To determine whether the porcine blood CD1^−^ cDC population shares this property, DC populations from two PRRSV-immune pigs were sorted, pulsed with either a synthetic peptide bearing previously identified CD4 and CD8 epitopes^52^ or inactivated PRRSV, and the stimulation of IFN-γ responses from autologous CD4 and CD8 T cells was assessed by flow cytometry (Fig. 5B and Supplementary Figure S2). Using the synthetic peptide, we observed that both cDC populations were more effective at stimulating CD4 T cell IFN-γ responses compared with pDC (p < 0.05); a similar trend was observed for CD8 T cells, although without statistical significance. Notably, there was no significant difference between the stimulatory properties of the two cDC populations for either T cell population (Fig. 5B). However, when cultured with inactivated virus, the CD1^−^ cDC showed a significant increase in their capacity to stimulate CD8 T cells compared with CD1^+^ cDC and pDC (p < 0.05). Although there was no significant difference between the cDC populations in their ability to stimulate virus-specific CD4 IFN-γ responses, CD1^−^ cDC were superior to both pDC and monocytes.

Finally, we compared the ability of porcine blood DC populations to stimulate primary allogeneic T cell responses using an MLR. In humans, CD1c^+^ cDC stimulate the strongest allogeneic MLR responses^53^, while others have demonstrated an equal ability in murine liver-derived cDC populations^54^. We found that CD1^−^ cDC induced significantly stronger proliferation of allogeneic T cells than CD1^+^ cDCs, pDCs and monocytes at 1:2, 1:6 and 1:18 (p < 0.0001) and 1:54 (p < 0.01) stimulator:responder cell ratios. CD1^+^ cDCs were also better at stimulating allogeneic T cells than monocytes and pDCs at a 1:2 ratio (p < 0.0001) and also at a 1:6 ratio compared to pDCs only (p < 0.01) (Fig. 5C). Interestingly, pDC showed very limited ability to stimulate allogeneic T cell proliferation.

### Porcine blood cDC populations show distinct gene expression signatures

**Figure 6:**
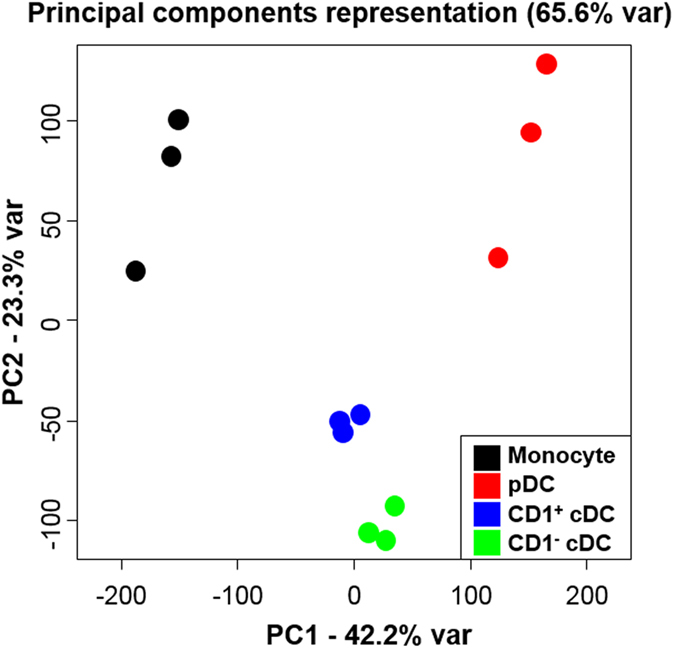
Gene expression profiling of porcine blood DC and monocytes. Principle component analysis (PCA) analysis of the isolated cell populations Monocytes (black), pDC (red), CD1^+^ cDC (blue) and CD1^−^ cDC (green) clusters showing two principle components representing 65.6% of total gene variation. Data for sorted blood DCs subsets and monocytes from three pigs are presented.

The data above show that the two porcine blood cDC populations identified are phenotypically and functionally distinct, and share characteristics with the cDC1 or cDC2 populations defined in other mammalian species. However, due to limits in the availability of porcine reagents compared to other species such as humans and mice, only a limited number of markers could be studied at the protein level. To explore a broader range of DC markers, we compared the porcine DC populations at the transcriptome level. This also enabled us to determine if the DC populations share a common gene expression signature as has been recently described for other species^24^,^33^,^50^,^55^,^56^. Employing a custom made NimbleGen 12 × 135 K porcine array spanning a total of 19,351 genes, we investigated the gene expression profile of the FACS sorted CD1^−^, CD1^+^ and pDC populations from three separate pigs and compared these with monocytes from the same pigs. Principle component analysis of the gene expression analysis of cell subsets showed a separation between CD14^+^ monocytes on the one hand with the three blood DC populations (pDC, CD1^−^ cDC and CD1^+^ cDC) on the other hand, along the first axis representing the major source (42%) of variability within the dataset (Fig. 6). Moreover, on the second axis, still representing 23% of the variability of the dataset, porcine CD1^−^ and CD1^+^ cDC were very close and clearly separated from both CD14^+^ monocytes and pDC. This near proximity supports the notion that these populations are highly enriched for cDC, being less similar to both monocyte and pDC. Three hundred and ninety six genes were significantly differentially expressed between CD1^−^ and CD1^+^ DC, 133 genes were expressed at a higher level in CD1^−^ cDC while 263 genes were expressed at a higher level in the CD1^+^ cDC (Supplementary Table S1). Of the 263 genes up-regulated in CD1^+^ cDC, 7 genes had been previously reported to be upregulated in the cDC2 subset in other species (Table 1). These were genes encoding C-type lectins CD206 (MRC1) and CD302 (CLEC13A/DCL-1); TLR-1, -4 and -5; IL-10 and the IFN-stimulated gene IFIT3 (Table 1). Furthermore, 10 genes up-regulated in CD1^−^ cDC- were also highly expressed in cDC1s from other species. The majority of these genes encoded membrane proteins (XCR1, CLEC12a, CD36, CD59, ANPEP and SEMA4f) although genes expressing cytosolic (PLEKHA5, FKBP1b-like and OXCT1) and secreted proteins (MMP9) were also identified (Table 1). Analysis of the genes for the nine TLRs showed evidence of increased expression of TLR-3, -7, -8 and -9 in the CD1^−^ DC population (although this was not found to be statistically significant) and an increased expression of TLR-1, -2, -4 and -5 in the CD1^+^ DC population (Supplementary Table S2). These expression data are consistent with the observed stimulation of the same cDC populations with poly(I:C) (TLR3), CpG (TLR9) (both CD1^−^ cDC) and LPS (TLR4) (CD1^+^ cDC) as shown in Fig. 4.

Finally, gene set enrichment analysis (GSEA) was employed to compare the transcriptomic signatures of the porcine CD1^−^ and CD1^+^ cDC populations with publically available data sets corresponding to the human CD141^+^ and CD1c^+^ and mouse CD8α^+^ and CD11b^+^ populations (Table 2; See Supplementary Figure S3 and S4 for CD1^−^ and CD1^+^ cDC enrichment plots, respectively, and Supplementary Table S3 for the human and murine datasets analysed). Monocytes were selected as a reference population due to their shared myeloid lineage with DC and their orthology across species^57^. The porcine blood CD1^−^ cDC transcriptomic signature was enriched in mouse cDC1 (ES = 0.50, FDR = 0.299) and statistically significantly enriched in human cDC1 (ES = 0.46, FDR = 0.002), when compared to classical monocytes (cMo) from the same species. Similarly, the porcine blood CD1^+^ cDC transcriptomic signature was significantly enriched in both mouse cDC2 (ES = 0.53, FDR = 0.013) and also, to a greater extent, in human cDC2 (ES = 0.49, FDR = 0.005). Conversely, the porcine blood CD14 ^+^ monocyte signatures were enriched in the human and mouse cMo when compared to cDC1 or cDC2.

## Discussion

This study has provided a phenotypic and functional analysis of cDC from porcine blood. To the best of our knowledge, this study is the first to have rigorously isolated and studied porcine blood cDC2, and to have characterized them at the transcriptomic and functional levels. The notion that CD1 expression may define distinct subsets was supported by the differential expression of CD1c by human blood cDC2. The complement receptor CD11R3 served as a second discriminatory marker since it was expressed only by CD1^+^ cDC. Notably CD11R3 is described as the orthologue for CD11b^43^, which is expressed on the mouse cDC2. CD1^+^ cDC were also found to express higher levels of CD172a, previously reported in murine, ovine and human cDC2^33^. Lower levels of CD172a were expressed on CD1^−^ cDC population. This is in contrast to claims that cDC1 DC populations lack expression of CD172a^35^,^58^. This disparity is most likely due to the fact that these cells were isolated from blood as opposed to tissues. This is supported by the report of low expression of CD172a on cDC1 in human blood^59^.

Comparison of the porcine blood DC phenotypes with DC isolated from other tissues was beyond the scope of the present study although some comparisons with previously published reports can be made. Others have previously demonstrated that DC populations in skin and draining afferent lymph and the lung could be defined by expression of CD172a and CD163^35^,^37^. Transcriptomic and functional studies demonstrated that the CD163^low^ population expressed high levels of CD172a, were XCR1 negative, and shared gene expression with cDC2 suggesting they are the tissue resident equivalents of the CD1^+^ subpopulation described here. Likewise, the CD172a^−^ cells were the only population to express XCR1, therefore likely to resemble the cDC1 population described in human and mouse and the CD1^−^ population described here^36^,^37^.

The functional analyses further support an organised specialisation between the populations. The CD1^−^ cDC responded to PAMPs representing pathogen nucleic acids, poly(I:C) and CpG stimulating TLR 3 and 9 respectively with secretion of IFN-α and IL-12. In contrast, CD1^+^ cDC primarily responded to LPS stimulating TLR 4, with secretion of IL-10 and IL-8. These data suggest that CD1^−^ cDC are programmed to drive type 1 T cell responses to control intracellular pathogens whereas CD1^+^ cDC drive type 2 T cell responses and support antibody responses to extracellular pathogens. The differential expression of IL-12 and IL-10 implies that the populations may also have a direct and opposing influence on one another. Like CD1^−^ cDC, human CD141^+^ cDC were also found to be a prominent source of IL-12 and IFN-α after stimulation via TLR 3^60^. Similarly, in the mouse, higher levels of IL-12 and IFN-α were associated with CD8α^+^ cDC1s following stimulation via TLR 3 and 9 relative to CD11b^+^ cDC2s^8^,^10^. Consistent with the CD1^+^ cDC2 responses observed, CD1c^+^ cDC express TLR4^29^ and secrete IL-10, but not IL-12, in response to *E. coli*^61^. The microarray data also demonstrated increased expression of TLR4 together with TLR1, TLR2, and TLR5 on CD1^+^ DCs. There was a marginal increase in TLR3 and 9 on the CD1^−^ DCs as shown previously in mouse^8^ (not statistically significant). Notably, in the equivalent human blood CD141^+^ DCs, there was no expression of TLR9^29^.

Our studies demonstrate that CD1^+^ cDC were able to take up soluble antigen most effectively which is consistent with the increased rates of antigen uptake reported *in vivo* by mouse CD11b^+^ cDC2 localised in the splenic marginal zone compared to other splenic DC^62^. However, more recently it has been shown that mouse and human DC subsets demonstrate a similar capacity to take up soluble and particulate antigen^29^,^51^. Collectively, these data suggest that an ability to take up antigen is not a reliable way of delineating cDC subsets. The superior ability of CD1^−^ cDC to stimulate allogeneic T cells is in line with the corresponding ovine CD26^+^ cDC subset^33^. CD1^−^ cDC were also superior in their ability to cross-present viral antigen to CD8^+^ T cells which is a hall mark of CD8α^+^ DC function^51^,^63^ although this was only observed when cells were pulsed with whole virus and not when a synthetic 28mer peptide was applied. It is possible that the limited processing requirements of the peptide reduced the cellular requirements for cross-presentation. Overall, these functional studies provide evidence of orthology between cDC populations of humans, mice, sheep and pigs. GSEA analysis showed a conserved transcriptome signature between the pig CD1^−^ and CD1^+^ cDC populations and publically available mouse splenic CD8α^+^ and CD11b^+^ cDCs and human CD141^+^ and CD1c^+^ populations. That these subpopulations of cDC share a common genetic signature has been reported previously in sheep, mouse and human^24^,^33^,^50^,^55^,^64^,^65^. A recent study confirmed orthology between the blood cDC1 population in pig with mouse and human equivalents, although the authors were unable to make any claims on the cDC2 population^56^. In this study, cDC1 were identified based on CADM1 expression^27^ rather than an absence of CD1 expression as employed here. Our preliminary assessment of CADM1 expression on DC subsets showed that whilst CADM1 expression was highest on CD1^−^ cDC it was not uniformly so (Supplementary Figure S5). A more recent study reported the use of fluorescently-labelled recombinant XCL1 to identify cDC1 in porcine blood^66^. Given that XCR1 is an exclusive marker for the cDC1 population across species, we sought to establish the expression of XCR1 by the CADM1^negative^, CADM1^dim^ and CADM1^high^ cells which constituted the CD1^−^ cDC population (Supplementary Figure S5). Using monocytes as a reference population, RT-qPCR demonstrated negligible levels of XCR1 on CD1^+^ DCs while relatively high levels were expressed on the CADM1^high^ cells. Interestingly, XCR1, was also detected on the CADM1^negative^ and CADM1^dim^ cells albeit at a lower level than observed on the CADM1^high^ cells, in contrast to FLT3 which was expressed at similar levels. It may be hypothesised that the diverse expression of CADM1 and XCR1 on CD1^−^ cDC reflects a heterogeneous population of cDC1 at differing stages of maturation.

In summary, this study has demonstrated the existence of two functionally distinct cDC subsets in pig blood that are aligned with the existing definition of DC populations by the IUIS and current understanding of cDC populations in other mammalian species. The ability to readily enrich these cell populations from peripheral blood provides a new model system to investigate DC plasticity and interactions with pathogens, including a number of important myelotropic viruses, and vaccines.

## Methods

### Animals

All animal work was approved by the Animal and Plant Health Agency Ethics Committee and all procedures were conducted in accordance with the UK Animals (Scientific Procedures) Act 1986 under Project Licences PPL 70/7057 and 70/7209. Blood samples were collected from healthy Large White/Landrace cross-bred pigs, 6–24 months of age, by venopuncture of the external jugular vein. In selected experiments, blood was collected from pigs rendered immune to porcine reproductive and respiratory syndrome virus (PRRSV) by repeated intranasal inoculation with the attenuated genotype 1 PRRSV strain Olot/91^52^.

### Dendritic cell and monocyte isolation from porcine blood

PBMC were isolated from 200–500 ml of heparinised blood by density gradient centrifugation as previously described^67^. PBMCs were suspended in Dulbecco’s PBS without Mg^2+^ and Ca^2+^ (Life Technologies, Paisley, UK) supplemented with 2% FBS (Autogen Bioclear, Calne, UK) (PBS/2%FBS) and counted using a volumetric flow cytometer (MACSQuant Analyzer, Miltenyi Biotec, Bisley, UK). To deplete/isolate monocytes, PBMC were incubated with mouse anti-human CD14 microbeads (IgG2a; 10 μl/10^7^ cells; Miltenyi Biotec) for 15 min at 4 °C, washed twice (520 *g* for 10 min) passed through a 100 μm cell strainer (BD Biosciences, Oxford, UK), and applied to pre-equilibrated LD columns as indicated by the manufacturer (Miltenyi Biotec). The CD14^+^ cells were purged from the LD column in 3 ml of PBS/2% FBS with 2 mM EDTA (MACS buffer) and applied to an LS column. Essentially, DC were enriched using methods similar to those described previously^68^. Briefly, 10 μg/10^8^ cells of anti-porcine CD172a mAb (IgG2b, clone 74–22–15 A, Washington State University Monoclonal Antibody Center (WSUMAC), Pullman, USA) was added to the CD14 depleted fraction of cells for 30 minutes at 4 °C. After washing, cells were incubated with anti-mouse IgG microbeads (10 μl/10^7^ cells, Miltenyi Biotec) as described above and CD172a^+^ cells were isolated by applying the cells to an LS column (Miltenyi Biotec). Both CD172a^+^ and CD14^+^ cells were purged from the LS columns in 5 ml of MACS buffer and counted as above. To enable flow cytometric sorting of DC subsets from the CD14^−^CD172a^+^ enriched population, cells were stained with IgG1 mAbs directed to lineage (lin) markers (CD14, clone CAM36A, WSUMAC; CD3, clone 8E6, WSUMAC; CD8α, clone PT3613, WSUMAC; and CD21, clone B-Ly4, BD Biosciences), CD14, CD3 and CD8 mAbs were applied at a final concentration of 20 μg/10^8^ cells and CD21 mAb was used at 10 μg/10^8^ cells. After 30 min incubation at 4 °C, cells were washed and stained with PE-conjugated rat anti-mouse IgG1 secondary antibody (BD Biosciences). After washing, cells were stained with CD172a mAb conjugated to Alexa Fluor-647^®^ using the APEX™ Antibody Labelling Kit (Life Technologies) (10 μg/10^7^ cells), CD1-FITC mAb (2.5 μg/10^7^cells; clone 76-7-4, Southern Biotec, Cambridge Bioscience, Cambridge, UK) and CD4-PerCP™-Cy5.5 mAb (0.4 μg/10^7^cells, clone 74-12-4, BD Biosciences). In addition, 2.5 × 10^5^ cells were stained with each mAb individually to serve as single colour compensation controls. Cells were washed, suspended in RPMI-1640 medium supplemented with 10% FBS at 1 × 10^7^ cells/ml and sorted using a MoFlo^®^ Astrios™ Cell Sorter (Beckman Coulter, High Wycombe, UK). DC were gated as singlet, CD172a^+^ lin^−^ cells, and sorted into CD1^−^CD4^−^ cDC, CD1^+^ CD4^−^ cDC and CD1^−^CD4^+^ pDC populations. Any contaminating monocytes were excluded by gating out CD172a^hi^ events and lineage positive cells. Immediately after sorting, the cells were checked for purity and counted by flow cytometric analysis on a MACSQuant Analyzer (Miltenyi Biotec). To assess DC subsets without prior enrichment, PBMC were stained with the mAbs as described above but with the inclusion of CD14 PE-Texas Red (clone Tük 4; Life Technologies) and analysed using a BD Fortessa flow cytometer (BD Biosciences).

### Flow cytometric analysis of porcine blood DC subsets and monocytes

The sorted DC subsets and monocytes were seeded at 5 × 10^4^/well and stained with 10 μl of porcine MHC class II-DR (IgG1, clone 2E9/13), CD11R3 (CD11b-like; IgG1 clone 2F4/11), CD16 (IgG1, clone G7) mAbs (all Bio-Rad, Oxford, UK) and huCD152-muIg fusion protein (IgG2a, Enzo Life Sciences, Exeter, UK) conjugated to R-PE using Zenon^®^ Mouse IgG Labelling Kits (Life Technologies), and with biotinylated polyclonal anti-huCD83 antibody (R&D Systems, Abingdon, UK). Non-reactive antibodies matched by host and isotype were included at equivalent concentrations as controls. After 30 min at 4 °C the cells were washed twice with 200 μl/well of PBS/2% FBS and centrifuged as above. In the case of biotinylated CD83 antibody, streptavidin-PE (eBioscience, Hatfield, UK) was added (50 ng/well) and incubated for a further 20 min at 4 °C. All wells were washed twice and then cells were fixed by addition of 200 μl of CellFIX (BD Biosciences) and a minimum of 2.5 × 10^4^ cells were analysed on a MACSQuant Analyzer flow cytometer. To assess CADM1 expression, CD14 depleted, CD172a enriched cells were labelled as described above with the addition of anti-CADM1 mAb (clone 3E1, Caltag Medsystems, Buckingham, UK) and then detected with biotinylated anti-chicken IgY antibody (Stratech Scientific, New Market, UK) followed by streptavidin-Brilliant Violet 605 (BioLegend, London UK).

### Assessment of cytokine responses to TLR agonists

TLR agonists were diluted in cRPMI; LPS from *E. coli* K12, high-molecular weight polyinosinic-polycytidylic acid (poly(I:C)) (both 20 μg/ml) and class B CpG oligonucleotide ODN2007 (10 μM) (all from Invivogen, Source Biosource, Nottingham, UK) and applied to DC and monocytes seeded at 5 × 10^4^/well in triplicate wells. To serve as a negative control, 100 μl of cRPMI was added to an additional three wells. Recombinant porcine IL-3 was added at a final concentration of 10 ng/ml to all wells containing pDC in these and subsequent experiments. Following incubation for 18 h at 37 °C in a humidified 5% CO_2_ atmosphere, cell-free culture supernatants were removed and stored immediately at −80 °C for subsequent cytokine analysis. Culture supernatants were assessed for cytokine content using the Porcine Cytokine 1 Ciraplex™ Chemiluminescent Assay Kit (Aushon, Billerica, USA) and IL-12 ELISA (Porcine IL-12/IL-23 p40 DuoSet; R&D Systems, Abingdon, UK) according to the manufacturers’ instructions.

### Mixed leukocyte reaction

Sorted blood DC subsets and monocytes were adjusted to 2.5 × 10^5^ cells/ml in cRPMI and a three-fold dilution series of each population was prepared. Allogeneic PBMCs were added (5 × 10^5^ cells/well) at responder to stimulator cell ratios ranging from 2:1 to 162:1. Pokeweed mitogen (Sigma, Poole, UK) and cRPMI were added to wells containing only PBMC as positive and negative controls, respectively. After 72 h incubation at 37 °C in a humidified 5% CO_2_ atmosphere, cells were pulsed with 1 μCi/well ^3^H-thymidine (GE Healthcare, Little Chalfont, UK) and incubated for a further 24 h. Cells were harvested onto filter mats using a Harvester 96 Mach III (TomTec Inc, Hamden, USA) and ^3^H-thymidine incorporation measured by addition of 25 μl/well Microscint O and counting on a MicroBeta^2^ Plate Counter (both Perkin Elmer, High Wycombe, UK).

### Endocytosis/phagocytosis assay

Enriched blood DC or sorted monocytes were suspended in cRPMI and seeded at 5 × 10^4^ cells/well in round-bottom 96-well plates. Cells were pulsed with either 1.25 μg/well particulate antigen in the form of Alexa Fluor-647^®^-conjugated ovalbumin (Life Technologies) encapsulated in PLGA-nanoparticles^69^, or 2 μg/well of soluble Alexa Fluor-647^®^-conjugated ovalbumin (Life Technologies) to investigate phagocytosis and endocytosis, respectively. Cells were incubated at either 4 °C or 37 °C for 2 h, washed twice to remove free antigen, stained with CD1-FITC and CD4-PerCP-Cy5.5 mAb to discriminate DC subsets and uptake analysed by flow cytometry.

### Antigen processing and presentation assay

Blood DC subsets and monocytes were sorted from two PRRSV immune pigs and 1 × 10^5^ cells were pulsed in triplicate with 1 μg/ml synthetic 28mer peptide bearing previously identified CD4 and CD8 T cell epitopes from PRRSV^52^ or 10^5^ TCID_50_ equivalent dose of heat-inactivated (56 °C, 1 hr) PRRSV-1 Olot/91 strain. cRPMI or an equal volume of clarified cryolysate of MARC-145 cells (mock virus antigen) was added as a negative control for peptide and virus stimulations, respectively. Following an incubation at 37 °C for 2 h, cells were washed twice as above, and DC/monocyte depleted autologous PBMC (5 × 10^5^ cells/well) and GolgiPlug, (0.2 μl/well; BD Biosciences) were added to each well. Cells were then incubated 37 °C for a further 18 h before assessment of CD4^+^ and CD8^+^ T cell IFN-γ responses by flow cytometric analysis as previously described^67^.

### Gene expression microarray analysis

All kits were used according to the manufacturer’s instructions. Sorted blood DC subsets and monocytes were washed in cRPMI and 0.5–1 × 10^6^ cells collected by centrifugation (900 *g*, 3 min). The supernatants were removed and cells snap-frozen in liquid nitrogen and stored at −80 °C until RNA was extracted using an RNAqueous micro Kit (Life Technologies). The Ovation PicoSL WTA System v2 kit (NuGEN, Leek, The Netherlands) was used to amplify cDNA from 50ng total RNA. The MinElute Reaction Cleanup Kit (Qiagen) was used to purify cDNA, and 1 μg was then labelled using a one-color DNA labelling kit (NimbleGen, Madison, USA). For each sample, 4 μg labelled cDNA was hybridised to a custom NimbleGen 12 × 135 K porcine array designed using the *Sus scrofa* 10.2 genome build and incorporating a total of 19,351 genes, each represented on the array by a set of six different probes (116,106 probes in total). The microarray also contains a large number (24,179) of random probes. Hybridised arrays were scanned at 2 μm resolution on a microarray scanner (Agilent, Wokingham, UK). Microarray images were processed using DEVA v1.2.1 software to obtain a pair report containing the signal intensity values for each probe. The raw intensity values were corrected for background by subtracting the median intensity values of the 20 nearest neighbour random probes. To correct for differences in the overall intensity levels between slides robust multi-array normalisation was used. From this point, the expression analysis was assessed at probe level as well as gene level. At the probe level, differential probe intensity between any two given cell types were identified using the Limma package^70^ with the p-values adjusted for multiple testing using the Benjamini and Hochberg’s method. Using the normalised probe intensity data matrix, the two first principal components (65.5% of the cumulative variability in the data set) were used to visualise the overall gene expression relationship between the samples^71^ The PCA analysis showed an acceptable agreement between the biological replicates and also a significant segregation between the sample conditions (Fig. 6). To calculate the gene expression level, the median polish algorithm was applied to the normalised probe intensity data matrix^72^. Differential gene expression tables were completed with information on the corresponding probes intensities; the number of probes with an adjusted p-value < 0.05 (as significant), 0.05–0.1 (as low significance) and >0.1 (as non-significant). When comparing gene expression between two cell types, a gene was considered for further analysis if: (1) at the gene-level, it showed a significant difference (adjusted p-value < 0.05) between cell types, (2) at the gene-level, the difference of expression between cell types was greater than 2-fold and (3) at the probe-level, no less than 4 of the probes showed significant differences between cell types. The raw microarray data (background-corrected signal) can be assessed at Gene Expression Omnibus (GEO accession GSE84029).

### Pairwise gene set enrichment analysis for cross-species comparison of porcine blood cDC subsets

To assess the orthology of the transcriptome of the porcine cDC populations with published datasets from equivalent human and murine cDC populations, Gene Set Enrichment Analysis (GSEA) was applied^73^. The published human and murine cDC transcriptomic datasets were obtained from GEO accessions GSE35459 and GSE15907, respectively (see Supplementary Table S3 for further details regarding the human and murine datasets analysed). The GSEA-P package was employed. As input gene sets, we generated the up- and down-regulated gene transcriptomic signatures of porcine blood CD1^−^ cDC or CD1^+^ cDC when compared to CD14^+^ monocytes. We then examined whether these transcriptomic signatures/gene sets were significantly enriched in the corresponding human or mouse cell type using pairwise comparisons between cDC1 or cDC2 versus CD14^+^ monocytes in each species. To generate the ranked gene lists for these species, the GSEA-P package was employed based on the entire data sets. The enrichment scores (ES) and their statistical significance (p) were calculated for the gene sets in each of the cell population comparisons. The risk of false positive enrichment was estimated using the false discovery rate (FDR, q) calculated upon performing 1,000 random permutations of classes^73^.

### Additional data analysis and statistics

Graphical and statistical analysis of non-array data was performed using GraphPad Prism 6 (GraphPad Software Inc, La Jolla, USA). Data was represented as means with standard errors (SEM). A two tailed unpaired t-test or a one-way analysis of variance (ANOVA) followed by Dunnett’s test was used and a p-value <0.05 was considered statistically significant.

## Additional Information

**How to cite this article**: Edwards, J. C. *et al*. CD1^−^ and CD1^+^ porcine blood dendritic cells are enriched for the orthologues of the two major mammalian conventional subsets. *Sci. Rep.* **7**, 40942; doi: 10.1038/srep40942 (2017).

**Publisher's note:** Springer Nature remains neutral with regard to jurisdictional claims in published maps and institutional affiliations.

## Supplementary Material

Supplementary Information

## Figures and Tables

**Table 1:** Differential gene expression in porcine blood cDC populations with orthology in other species.

Porcine cDC population	Gene name	Fold change^a^	P-value	Human, murine or ovine DC subset^b^
CD1^+^	MRC1	12.2	0.015	HuCD1c^50^
CD1^+^	TLR5	6.44	0.026	MuCD11b, HuCD1c^24^
CD1^+^	TLR4	6.33	0.075	HuCD1c^61^
CD1^+^	CD302	4.77	0.017	MuCD11b, HuCD1c^24^
CD1^+^	IFIT3	3.74	0.028	MuCD11b, HuCD1c^24^
CD1^+^	TLR1	3.44	0.021	MuCD11b, HuCD1c^24^
CD1^+^	IL-10	3.05	0.05	HuCD1c^61^
CD1^-^	XCR1	29.81	0.025	HuCD141, MuCD8α, OvCD26^33^,^55^
CD1^−^	ANPEP	23.3	0.023	HuCD141, MuCD8α, OvCD26^33^
CD1^−^	CD59	18.82	0.078	HuCD1c^65^
CD1^−^	MMP9	14.01	0.06	HuCD141^74^
CD1^−^	PLEKHA5	8.73	0.021	HuCD141, MuCD8α^24^
CD1^−^	SEM4f	8.66	0.014	HuCD141, MuCD8α^24^
CD1^−^	S100-z-like	8.62	0.058	MuCD8α^8^
CD1^−^	CD36	7.86	0.116	MuCD8α^25^
CD1^−^	IL12RB2	4.56	0.027	MuCD8α^33^
CD1^−^	ADAMDEC1	4.25	0.048	MuCD8α^8^
CD1^−^	CLEC12a	3.52	0.025	MuCD8α^64^
CD1^−^	FKBP11-like	3.02	0.012	HuCD141, MuCD8α^24^
CD1^−^	OXCT1	2.60	0.035	MuCD8α^8^

^a^Expression fold-change CD1^+^ vs. CD1^−^ cDC displayed as absolute values

^b^Genes whose expression has previously been reported to be associate with cDC subsets in mice, humans or sheep.

**Table 2:** Summary of the gene-set enrichment analysis of porcine blood cDC transcriptomes versus orthologous human and mouse cDC subsets.

Porcine blood cell type Gene sets	Pairwise comparisons between mouse or human cell types	Enriched in	Enrichment Score (ES)^a^	P - value^b^	False Discovery Rate (q)^b^
CD1^−^ vs CD14^+^ up	Human cDC1 vs CD14^+^ cMo	cDC1	0.46	0	0.002
CD1^−^ vs CD14^+^ up	Mouse cDC1 vs Ly6c^+^ cMo	cDC1	0.50	0.171	0.299
CD1^−^ vs CD14^+^ down	Human cDC1 vs CD14^+^ cMo	cMo^c^	−0.67	0.005	0.007
CD1^−^ vs CD14^+^ down	Mouse cDC1 vs Ly6c^+^ cMo	cMo	−0.59	0.113	0.266
CD1^+^ vs CD14^+^ up	Human cDC2 vs CD14^+^ cMo	cDC2	0.49	0.003	0.005
CD1^+^ vs CD14^+^ up	Mouse cDC2 vs Ly6c^+^ cMo	cDC2	0.53	0.021	0.013
CD1^+^ vs CD14^+^ down	Human cDC2 vs CD14^+^ cMo	cMo	−0.64	0	0.002
CD1^+^ vs CD14^+^ down	Mouse cDC2 vs Ly6c^+^ cMo	cMo	−0.55	0.025	0.057

^a^The ES is calculated out of a possible maximum of 1 and minimum of −1.

^b^Values of p ≤ 0.1 and q ≤ 0.25 are considered to indicate significant enrichment.

^c^Classical monocytes.
