# Supplementary material for: CD1− and CD1+ porcine blood dendritic cells are enriched for the orthologues of the two major mammalian conventional subsets
Source: Sci Rep. 2017 Jan 20;7:40942. doi: 10.1038/srep40942 (PMC5247722; doi:10.1038/srep40942)
Supplement: Supplementary Information [file srep40942-s1.pdf]

Supplementary Information

CD1^{-} and CD1^{+} porcine blood dendritic cells are enriched for the orthologues of the two major mammalian conventional subsets. Jane C. Edwards, Helen E. Everett, Miriam Pedrera, Helen Mokhtar, Emanuele Marchi, Ferran Soldevila, Daryan A. Kaveh, Philip J. Hogarth, Helen L. Johns, Javier Nunez-Garcia, Falko Steinbach, Helen R. Crooke, Simon P. Graham

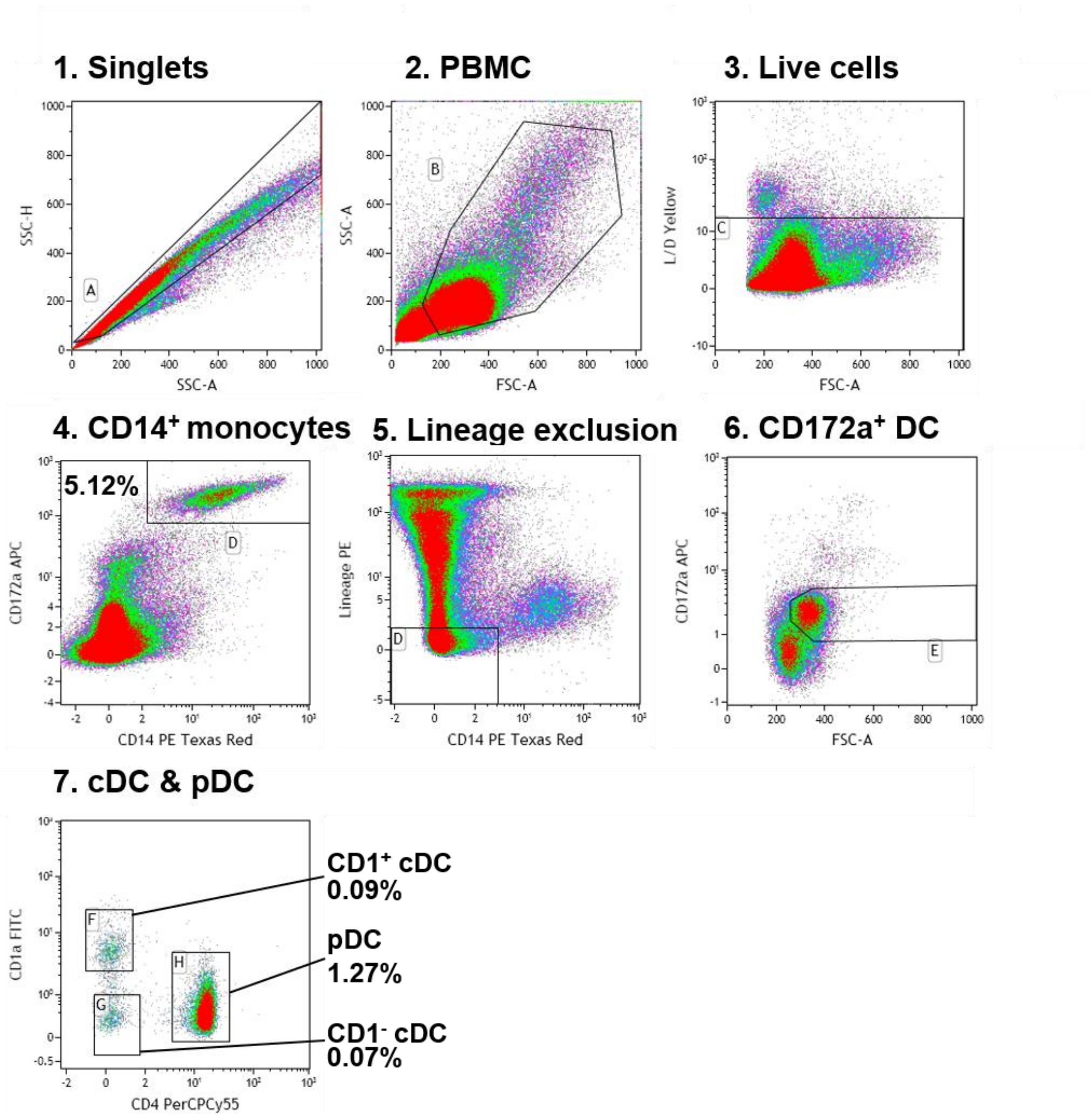

Supplementary Figure S1: Staining of porcine blood DC populations within PBMC. PBMC were isolated, stained with mAbs and analysed by flow cytometry. Illustrative dot plots show the gating strategy: Singlet (**1**), mononuclear (**2**), live cells (**3**) were first gated on. Monocytes were gated as $CD172a^{high}CD14^{+}$ cells (**4**). DCs were gated as $CD14^{-}$ lineage $marker^{-}$ (CD3, CD8α, CD21) (**5**), and low to moderate expression of $CD172a^{+}$ (**6**). $CD1^{-}$ cDC, $CD1^{+}$ cDC, and pDC were finally gated on differential expression of CD1 and CD4 (**7**). The proportions of monocytes, $CD1^{-}$ cDC, $CD1^{+}$ cDC, and $CD4^{+}$ pDC expressed as a % of live PBMC are presented.

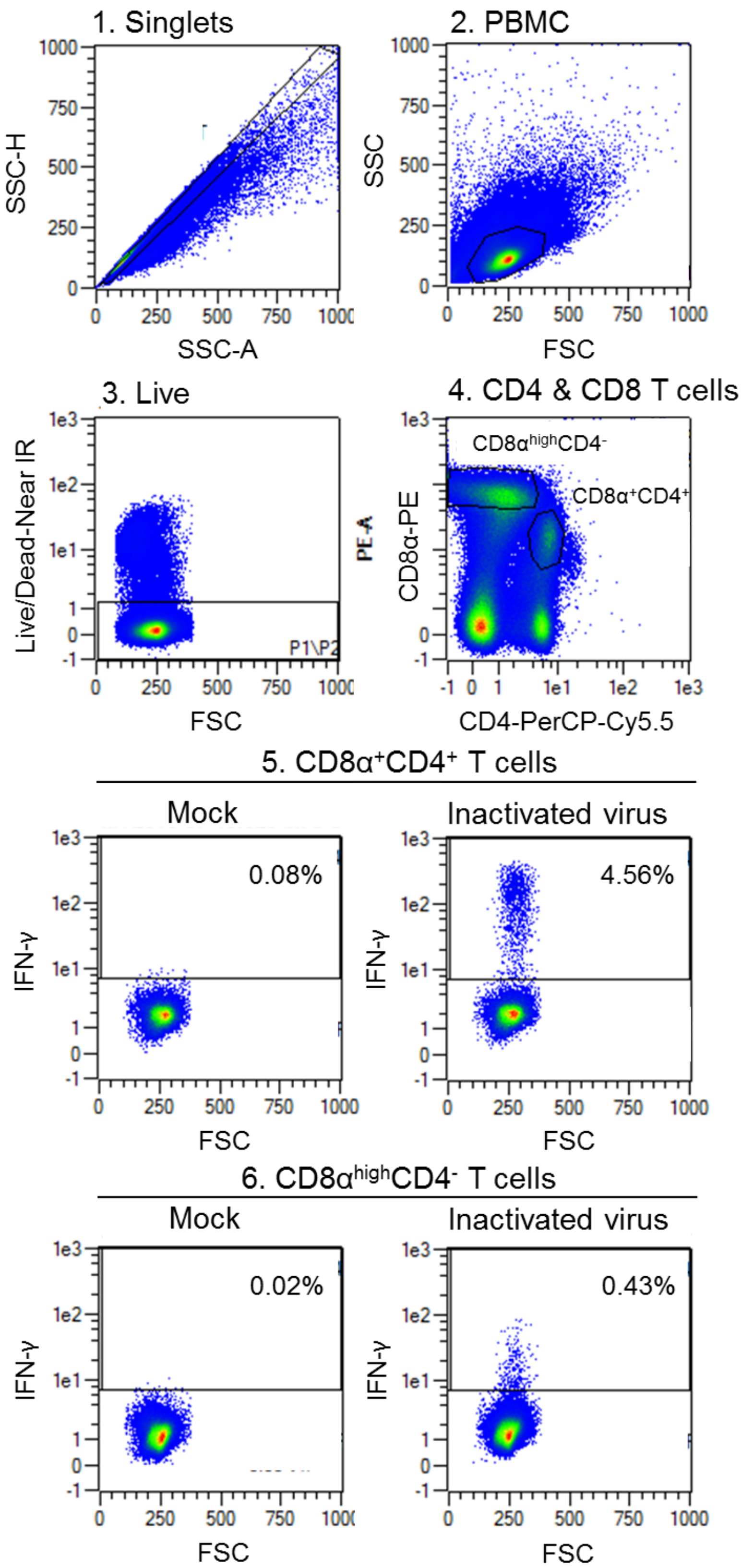

Supplementary Figure S2: Gating strategy to assess stimulation of antigen-specific CD4$^+$ and CD8$^+$ T cell IFN-γ responses by antigen-pulsed sorted blood DC populations and monocytes. Single cells were gated upon using SSC-A vs SSC-H (**1**) and then lymphocytes were gated upon using typical FSC vs SSC properties (**2**). Live/Dead stain$^-$ cells (**3**) were assessed for CD4 and CD8α expression (**4**) and the two populations of CD4 (CD4$^+$CD8α^{low}) (**5**) and CD8 (CD4$^-$CD8α^{high}) (**6**) T cells were assessed for IFN-γ expression. IFN-γ gates were set using corresponding biological negative controls.

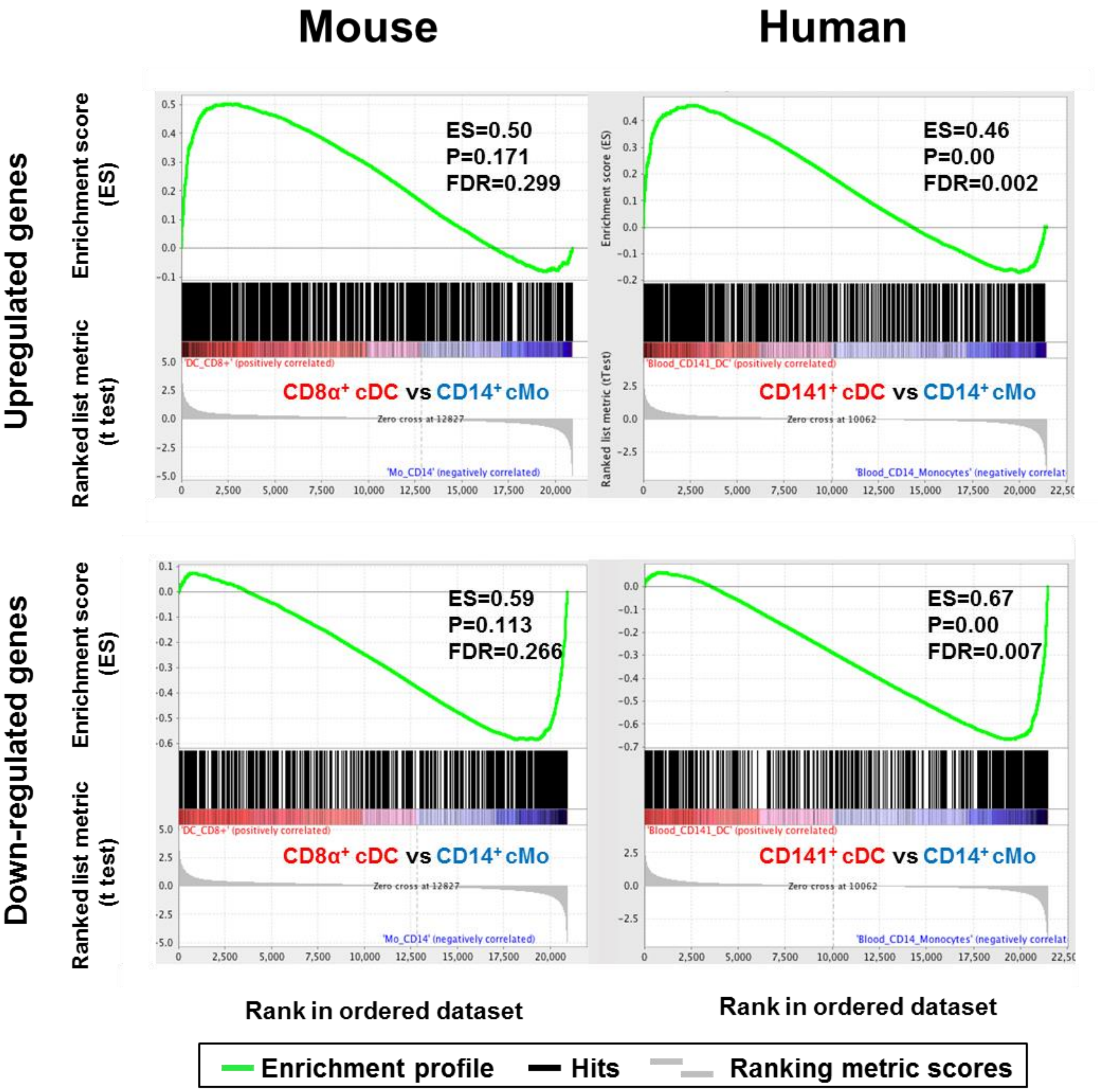

Supplementary Figure S3: Gene set enrichment analysis between human or mouse DC subsets and the porcine blood CD1^{-} cDC population. The gene sets used represent transcriptomic signatures of the blood porcine CD1^{-} cDC population as compared to CD14^{+} cMo. Enrichment of these gene sets were assessed in pairwise comparisons between cDC1 and cMo, both in mouse (column 1) and human (column 2). Results are shown for both up-regulated and down-regulated genes as i) enrichment scores (ES), ii) probabilities (p) and iii) false discovery rates (FDR).

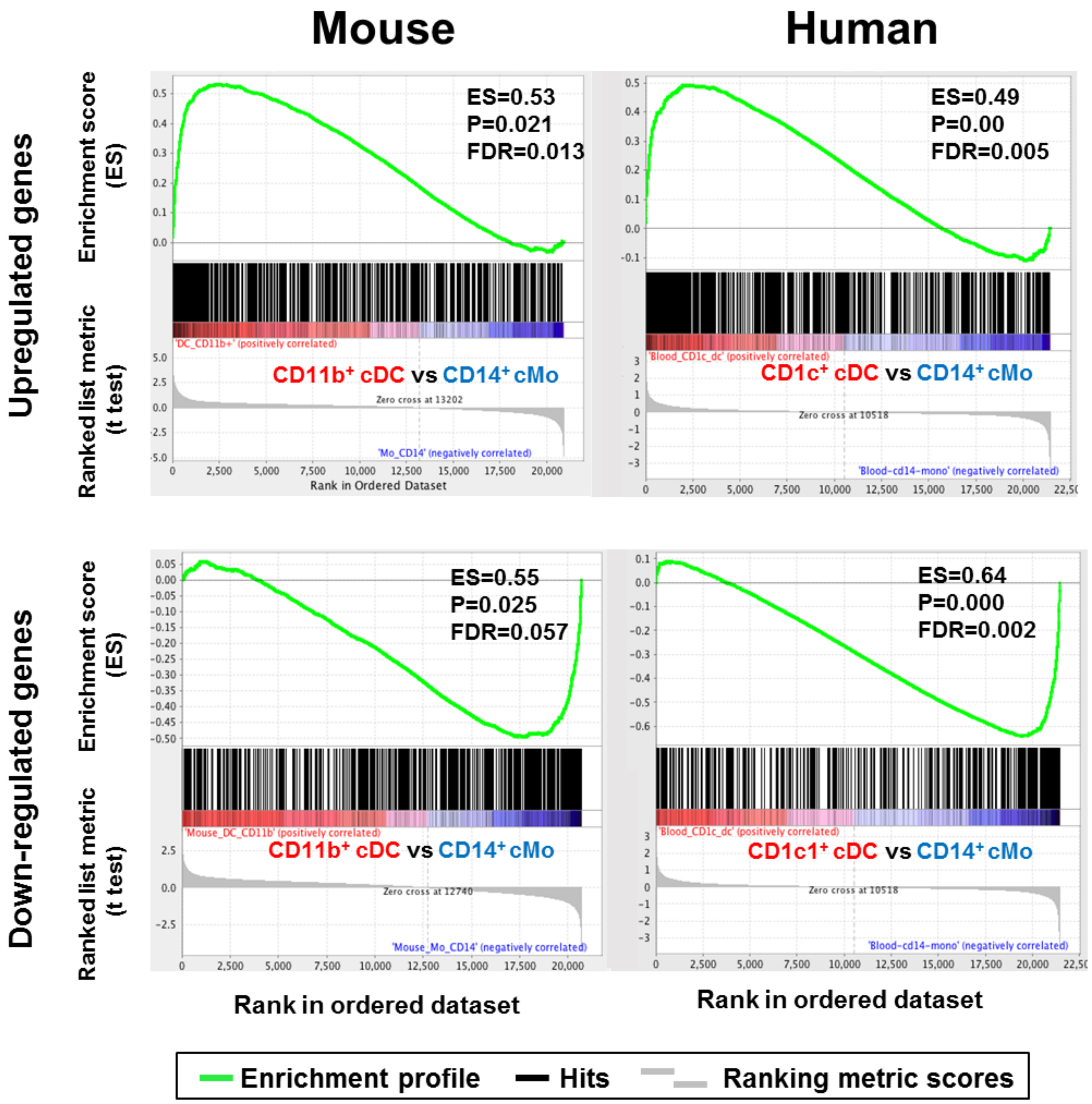

Supplementary Figure S4: Gene set enrichment analysis between human or mouse DC subsets and the porcine blood CD1^{+} cDC population. The gene sets used represent transcriptomic signatures of blood porcine CD1^{+} cDC as compared to CD14^{+} cMo. Enrichment of these gene sets were assessed in pairwise comparisons between cDC2 and cMo, both in mouse (column 1) and human (column 2). Results are shown for both up-regulated and down-regulated genes as i) enrichment scores (ES), ii) probabilities (p) and iii) false discovery rates (FDR).

Supplementary Table S1. Significantly differentially expressed genes between porcine blood CD1⁻ and CD1⁺ cDC populations.

SEQ_ID	Gene Symbol	Fold Change*	Adjusted P-value
0610007P14RIK101200001520	0610007P14RIK	-1.39	0.058047048
ABHD6101200017322	ABHD6	-1.42	0.034544199
ACYP1101200001717	ACYP1	2.65	0.015384981
ADAM22101200015177	ADAM22	-5.84	0.033825864
ADAMDEC1101200017859	ADAMDEC1	-4.25	0.048296433
ADCY6101200011174	ADCY6	-21.17	0.011325959
ADCY6101200011175	ADCY6	-25.08	0.011325959
ADIPOQ101200002644	ADIPOQ	-23.76	0.036470115
AMBN101200002312	AMBN	7.29	0.017263058
ANKS3101200009725	ANKS3	-2.17	0.027089529
ANPEP101200002551	ANPEP	-23.3	0.023381299
APBA1101200007053	APBA1	-3.1	0.040490047
AQP3101200000708	AQP3	-14.03	0.033059752
ARHGAP21101200015826	ARHGAP21	-2.83	0.049367754
ARMC4101200015819	ARMC4	9.73	0.023381299
ASCT1101200010180	SLC1A4	-11.3	0.062519114
ATP13A1101200008661	ATP13A1	-2.76	0.015384981
ATP1B1101200000022	ATP1B1	-3.13	0.037686536
ATRNL1101200006021	ATRNL1	-45.16	0.048296433
AUH101200005579	AUH	1.7	0.044928669
BMPR1A101200001935	BMPR1A	5	0.023625186
BTAF1101200003338	BTAF1	-1.75	0.027241698
CCDC85A101200010214	CCDC85A	-3.63	0.024442551
CCNI101200014157	CCNI	1.44	0.043288053
CD180101200002631	CD180	2.55	0.016396755
CD302101200000720	LY75	4.77	0.016801473
CD34101200002361	CD34	-5.07	0.045339262
CD36101200000404	CD36	-7.86	0.115941477
CD59101200002444	CD59	-18.82	0.077635271
CEP110101200003602	CNTRL	-1.7	0.020517657
CEP152101200006637	MYEF2	-3.53	0.026029128
CLEC7A101200001330	CLEC7A	7.56	0.024686261
CLIC5101200001886	CLIC5	-17.22	0.015916516
CMKLR1101200000882	CMKLR1	-6.21	0.09911183
CORO1C101200005957	CORO1C	-1.63	0.060596219
COX7A1101200002685	COX7A1	-2.04	0.033881089
CPN1101200006013	CPN1	5.94	0.016228823
CRHBP101200001823	CRHBP	-11.71	0.078346051
CTSD101200000272	CTSD	-14.28	0.025306219
DDHD1101200006910	DDHD1	2.19	0.035605036
DMD101200000188	DMD	-9.14	0.058265406
DNMBP101200005996	DNMBP	-4.7	0.023062814
DOCK5101200017871	DOCK5	-3.77	0.046010599
DPP4101200002531	DPP4	-7.05	0.011325959

ECE1101200012748	ECE1	-4.61	0.025306219
EDN1101200002157	EDN1	-3.34	0.015384981
EPHA4101200001156	EPHA4	-11.75	0.083673049
EPHB4101200009464	EPHB4	-16.88	0.015384981
EPHX2101200000051	EPHX2	-6.49	0.017958814
FABP5101200000300	FABP5	4.28	0.015584013
FDFT1101200001531	FDFT1	-1.38	0.070677239
FECH101200001561	FECH	-1.68	0.059392883
FLNB101200017320	FLNB	-1.72	0.043486815
FRMPD4101200019962	FRMPD4	-4.08	0.018830335
FRY101200005092	FRY	-15.52	0.044385917
FRY101200005095	FRY	-10.29	0.061873285
GAB1101200014237	GAB1	-11.45	0.061755764
GALNT2101200004897	GALNT2	-4.55	0.023062814
GALNT3101200018490	GALNT3	5.63	0.019158591
GALNT7101200004069	GALNT7	-1.85	0.020011183
GPCPD1101200019282	GPCPD1	2.12	0.027387323
GPR126101200006289	GPR126	9.38	0.024686261
GRN101200000362	GRN	-6.91	0.052326608
GUCY1A3101200000195	GUCY1A3	-12.43	0.062157359
GUCY1B3101200000194	GUCY1B3	-11.56	0.115893429
HDC101200003415	HDC	-2.37	0.02949651
HDLBP101200018853	PASK	-4.24	0.016801473
HIP1101200009492	HIP1	-11.46	0.033513528
IFI16101200005864	IFI16	2.84	0.013229164
IFIT1101200005536	IFIT1	3.33	0.041996718
IFIT3101200001928	IFIT3	3.74	0.027699756
IFIT5101200003762	IFIT5	3.5	0.013343804
IGF1R101200002446	IGF1R	-6.32	0.05608771
IL10101200002316	IL10	3.05	0.04953808
IL12RB2101200002372	IL12RB2	-4.56	0.027089529
IL6ST101200000490	IL6ST	-3.55	0.030834869
IQGAP2101200008819	IQGAP2	-2.26	0.044926667
ISG15101200001042	ISG15	-4.13	0.017263058
ITGA5101200003360	ITGA5	-4.82	0.025634308
ITGAV101200000466	ITGAV	-2.94	0.022109523
ITGB3101200002277	ITGB3	-3.44	0.027089529
JUP101200002597	JUP	-12.69	0.015384981
KCNQ5101200003293	KCNQ5	2.2	0.047244674
KIF7101200004710	KIF7	-3.44	0.015384981
KLK1101200000078	KLK1	3.38	0.045902343
LDLR101200001970	LDLR	-10.3	0.062162498
LINGO1101200005842	LINGO1	-8.71	0.011325959
LMO3101200000740	LMO3	20.44	0.030232828
LOC100037944101200004155	CHI3L2	7.16	0.024686261
LOC100151817101200006765	LRRK1	-2.43	0.020517657
LOC100151849101200004070	ARV1	2.52	0.015584013
LOC100151849101200018070	ARV2	2.46	0.015384981
LOC100152196101200004816	UBE2W	1.74	0.030425785
LOC100152360101200003994	CCDC68	-9.92	0.012427897
LOC100152559101200004650	RGS10	1.83	0.030479518
LOC100153079101200003475	TESPA1	-2.14	0.027554787
LOC100153623101200005510	SORBS3	-8.97	0.036800319

LOC100154285101200004242	CRIP3	3.64	0.028794626
LOC100154289101200004203	FAM189A2	-13.91	0.037537515
LOC100154395101200004433	NOV	-15.57	0.030490282
LOC100154885101200017820	PIGP	2.52	0.020403291
LOC100155079101200004165	PTPRE	-3.83	0.016396755
LOC100155280101200005734	RHOBTB2	-3.62	0.025294663
LOC100155473101200004945	DOCK1	-3.55	0.047244674
LOC100155620101200001210	SLC19A2	3.42	0.057884637
LOC100155717101200003778	C14H10ORF58	-1.65	0.035768772
LOC100155802101200002986	C1ORF54	-6.62	0.073947964
LOC100155802101200010864	C1ORF54	-9.61	0.043486815
LOC100155807101200017886	GALNT7	-2.01	0.035605036
LOC100156110101200003641	CXHXORF21	1.97	0.023438853
LOC100156189101200003589	KIAA1598	1.91	0.046747782
LOC100156195101200002903	C1ORF51	-5.93	0.011325959
LOC100156452101200002883	OLFML2B	-8.6	0.012427897
LOC100156482101200003656	SORBS3	-8.99	0.02896391
LOC100156545101200002785	AACS	-3.63	0.033564886
LOC100156777101200005709	CASP7	1.93	0.021796586
LOC100156890101200004748	FCRL4	2.9	0.026029128
LOC100156943101200018194	EXOC6	2.37	0.025747186
LOC100157403101200005113	FRY	-10.08	0.054621558
LOC100157499101200002851	SLC38A6	3.45	0.017787831
LOC100157683101200006888	MYO9A	-1.61	0.041564094
LOC100157702101200002794	P2RY10	3.76	0.043482908
LOC100157741101200005508	TSPAN6	9.58	0.02676217
LOC100157926101200013230	RIOK1	-1.51	0.027089529
LOC100158069101200010782	FCRL3	4.29	0.024307928
LOC100158081101200005655	RHOBTB2	-4.57	0.035144963
LOC100158142101200004035	MYO5B	-4.27	0.012427897
LOC100158184101200016105	ABCC4	-2.91	0.017958814
LOC100302368101200001420	PRSS48	-4.3	0.03177933
LOC100510979101200017139	OSBPL10	-5.03	0.016396755
LOC100510998101200007213	STOM	-3.25	0.046235158
LOC100511070101200008849	HOMER1	9.35	0.02676217
LOC100511231101200018529	GPR155	-2.62	0.054621558
LOC100511528101200015284	GNAT3	-10.6	0.098633397
LOC100511735101200007367	RAPGEF1	-2.71	0.015384981
LOC100511847101200019775	CPED1	-6.08	0.017958814
LOC100512074101200017928	KDM2B	-3.18	0.021007558
LOC100512083101200006841	NEDD4L	-2.21	0.022109523
LOC100512100101200010343	LTBP1	-17.69	0.067026677
LOC100512142101200018979	ITGA1	-5.75	0.017412757
LOC100512157101200008786	MXD3	-2.25	0.02931515
LOC100512263101200006842	NEDD4L	-2.49	0.033059752
LOC100512387101200006641	HDC	-3.71	0.048106049
LOC100512418101200012794	CD164L2	-21.29	0.011325959
LOC100512472101200012026	GINS3	-2.7	0.025747186
LOC100512486101200015676	SLC35D2	2.16	0.036329209
LOC100512542101200013263	SOX4	-3.5	0.015384981
LOC100512584101200007713	PLA2G16	2.97	0.025289141
LOC100512642101200006843	PARP16	2.2	0.017723182
LOC100512663101200012931	OSBPL1A	1.63	0.056548

LOC100512751101200006387	COL19A1	-2.45	0.033564886
LOC100513231101200016629	ABCC3	-9.33	0.043486815
LOC100513309101200017436	TRH	-3.98	0.034406054
LOC100513336101200007787	CD5	-12.95	0.035768772
LOC100513564101200016482	JUP	-17.79	0.01519399
LOC100513576101200006608	CCPG1	1.39	0.059805115
LOC100513770101200006476	SLC16A10	2.95	0.047807623
LOC100513816101200016632	ABCC3	-6.16	0.024686261
LOC100513914101200008923	FER	-5.36	0.013343804
LOC100513927101200012033	NDRG4	-7.5	0.016106629
LOC100513964101200019918	YKT6	-2.55	0.015384981
LOC100514084101200006573	SPG21	2.54	0.020517657
LOC100514312101200015684	SLC35D2	2.16	0.024442551
LOC100514431101200018485	COBLL1	2.07	0.024161131
LOC100514482101200015240	AGR2	-14.15	0.016792364
LOC100514659101200013596	CMTM5	-5.24	0.025306219
LOC100514842101200012037	GPR97	-12.57	0.041996718
LOC100514999101200019980	PIR	-1.77	0.046747782
LOC100515004101200007154	MRPL50	2.52	0.020403291
LOC100515047101200015301	MAGI2	-39.79	0.017958814
LOC100515179101200019579	GALNT11	1.57	0.034331328
LOC100515238101200017321	DNASE1L3	-8.31	0.027866433
LOC100515290101200019981	C8H4ORF3	1.47	0.039077544
LOC100515322101200012944	CDH2	-13	0.106566329
LOC100515380101200012039	CCDC135	-18.19	0.02966989
LOC100515458101200019982	FRMPD4	-4.82	0.022821538
LOC100515621101200015970	FLT3	-2.53	0.035768772
LOC100515929101200017854	PDLIM2	-2.45	0.021284093
LOC100516517101200018144	BMPR1A	3.55	0.054021767
LOC100516606101200010293	COX7A2L	1.64	0.021247892
LOC100516653101200006259	CCDC170	-2.36	0.043602104
LOC100516699101200018145	GLUD1	1.45	0.055612365
LOC100516709101200019932	C7orf44	-2.69	0.027554787
LOC100516856101200011335	BLOC1S1	-2.4	0.035380202
LOC100516923101200014770	SYTL2	-8.24	0.075473394
LOC100516938101200017782	GBE1	1.91	0.026675588
LOC100516966101200010175	MEIS1	-19.84	0.043803792
LOC100517152101200010176	MEIS1	-23.2	0.039763108
LOC100517204101200019795	TFEC	3.65	0.011325959
LOC100517254101200019986	ZRSR2	-2.34	0.027089529
LOC100517293101200014315	CASP6	2.12	0.020828964
LOC100517581101200009535	TPST1	3.37	0.020517657
LOC100517759101200010895	PDE4DIP	4.46	0.041740051
LOC100517922101200006618	MYO5A	-1.97	0.017958814
LOC100517977101200018548	TTC30B	2.57	0.035605036
LOC100518083101200014386	HERC5	3.64	0.024161131
LOC100518100101200006218	MAP3K4	-1.72	0.022302468
LOC100518155101200018549	TTC30B	2.57	0.035620429
LOC100518164101200019936	GLI3	-6.85	0.011325959
LOC100518168101200020279	XKRX	2.34	0.02949651
LOC100518245101200010179	SLC1A4	-12.97	0.045328593
LOC100518696101200018453	FMNL2	-3.39	0.034544199
LOC100518838101200011804	TMCC3	-5.36	0.011325959

LOC100518873101200018705	TTLL4	-2.4	0.022021846
LOC100518910101200011684	IL17RA	-6.71	0.02949651
LOC100519058101200018656	FZD5	-3.01	0.017412757
LOC100519192101200011805	NUDT4	1.68	0.024686261
LOC100519195101200012130	LIN37	-2.15	0.049492359
LOC100519287101200020390	KLHL13	-4.43	0.048050148
LOC100519292101200007532	CCND1	-4.02	0.024161131
LOC100519322101200014022	SLAIN2	1.51	0.062275812
LOC100519324101200014327	ETNPPL	-3.6	0.011325959
LOC100519336101200017834	PDE9A	-3.52	0.023624306
LOC100519353101200008017	BBOX1	12.33	0.015384981
LOC100519369101200012686	DDI2	-2.28	0.057228911
LOC100519655101200010370	BRE	1.81	0.033513528
LOC100519703101200008820	F2RL1	-5.84	0.020517657
LOC100519888101200008821	S100Z	-8.62	0.057884637
LOC100519936101200006761	ASB7	2.04	0.035114782
LOC100520041101200014995	USP2	6.48	0.076588426
LOC100520051101200017942	C14H12ORF49	2.33	0.051280653
LOC100520105101200017790	C13H21ORF91	1.91	0.017958814
LOC100520135101200011572	CLEC12A	-3.52	0.025306219
LOC100520177101200008234	TRIP10	-2.67	0.041740051
LOC100520446101200015834	PIP4K2A	-1.48	0.047446945
LOC100520459101200017689	DTX3L	2.21	0.043367281
LOC100520477101200007751	RAB3IL1	-5.13	0.022061768
LOC100520765101200017637	CCDC50	-2.37	0.017263058
LOC100520765101200017638	CCDC50	-2.5	0.042534772
LOC100520836101200011230	GALNT6	-2.19	0.039368579
LOC100521253101200008828	IQGAP2	-2.15	0.105371178
LOC100521305101200006546	SMAD7	-2.17	0.036321944
LOC100521597101200006876	PAQR5	-12.2	0.080767001
LOC100521609101200008830	F2R	-12.09	0.110390209
LOC100521620101200010693	ADHFE1	5.18	0.023625186
LOC100521722101200010635	ZNF706	1.83	0.028252278
LOC100521879101200006716	PLCB2	-2.15	0.035902523
LOC100522015101200011234	GRASP	-7.1	0.011325959
LOC100522160101200016601	HOXB3	-6.36	0.017723182
LOC100522203101200015334	PDIA4	-2.06	0.023624306
LOC100522377101200012840	ZNF362	-2.93	0.021479572
LOC100522453101200014935	RBM7	1.73	0.051019249
LOC100522571101200017001	CD68	2.1	0.025634308
LOC100522740101200011935	TMEM170A	1.89	0.044928669
LOC100523170101200008439	RNASEH2A	-1.96	0.027321822
LOC100523288101200018569	HSP40	2.38	0.020517657
LOC100523384101200013536	KIF7	-5.87	0.027387323
LOC100523564101200015909	DIP2C	-12.26	0.063172504
LOC100523731101200010326	PKR	1.8	0.022109523
LOC100523738101200011763	CHPT1	8.16	0.023062814
LOC100523746101200015910	DIP2C	-8.08	0.023062814
LOC100523757101200019534	ARFGEF2	-2.29	0.017412757
LOC100523786101200011065	PACSIN2	-2.04	0.026317431
LOC100523816101200016395	ARHGAP27	-3.41	0.024590101
LOC100523885101200007409	TM2D3	1.92	0.043486815
LOC100523916101200015911	DIP2C	-6.06	0.062282399

LOC100523985101200015215	DYNC1I1	-3.27	0.02966989
LOC100524040101200016040	TSC22D1	-10.53	0.06883616
LOC100524195101200010204	B3GNT2	2.22	0.049136538
LOC100524220101200017536	P2RY14	-4.48	0.053763149
LOC100524325101200014285	PDE5A	-2.22	0.038837658
LOC100524412101200006457	SESN1	2.99	0.033881089
LOC100524600101200020043	PRRG1	5.59	0.035768772
LOC100524632101200013979	TLR1	3.13	0.045131281
LOC100524643101200016125	TMTC4	-2.43	0.035470968
LOC100524681101200012084	ZNF507	4.03	0.027089529
LOC100524761101200016086	KCTD12	-2.94	0.049663393
LOC100524777101200006373	ZUFSP	2	0.02676217
LOC100524793101200009357	CHST12	-8.46	0.022109523
LOC100524883101200014355	BANK1	3.7	0.040836509
LOC100524935101200011183	FKBP11	-3.02	0.012427897
LOC100524972101200007771	MS4A6A	-2.04	0.012427897
LOC100524984101200010699	MYBL1	10.44	0.018005152
LOC100524997101200013981	TLR6	3.54	0.015384981
LOC100525161101200010700	C4H8ORF46	5.76	0.028688338
LOC100525258101200018469	PLA2R1	-13.16	0.079531009
LOC100525477101200015097	SLC37A2	2.96	0.027387323
LOC100525523101200012980	IFI44	5.37	0.012427897
LOC100525541101200018276	CLRN3	-2.16	0.022608717
LOC100525682101200008261	INSR	-2.84	0.065086151
LOC100525723101200006286	ABRACL	1.36	0.035380202
LOC100525723101200006287	ABRACL	1.36	0.03678169
LOC100525752101200012434	POLD1	-3.7	0.023438853
LOC100525939101200014743	CLNS1A	1.86	0.026029128
LOC100526076101200017371	PROK2	9.51	0.02676217
LOC100526158101200008971	SNX24	2.84	0.033513528
LOC100526181101200015224	RPA3	2.07	0.04027082
LOC100526183101200016090	SLAIN1	-4.17	0.027699756
LOC100526184101200017138	OSBPL10	-3.87	0.023625186
LOC100526192101200019273	PSD3	-2.47	0.033564886
LOC396866101200002134	CSTA	-13.87	0.04027082
LOC396877101200008093	CFD	-2.08	0.027556578
LOC414384101200000039	TMEM251	4.12	0.015384981
LPIN2101200001204	LPIN2	-2.06	0.017958814
LRP8101200001897	LRP8	-7.56	0.029305563
MAN2C1101200002755	MAN2C1	-3.69	0.039892603
MAP3K6101200012793	MAP3K6	-6.32	0.011325959
MAT2B101200001218	MAT2B	1.52	0.023625186
MGLL101200001263	MGLL	-5.28	0.023062814
MGST1101200002574	MGST1	1.87	0.023625186
MMP16101200004154	MMP16	-7.88	0.029824954
MMP2101200002466	MMP2	-5.71	0.024858632
MMP9101200000282	MMP9	-14.01	0.060232765
MRC1101200015782	MRC1	12.2	0.015384981
MUDENG101200004903	AP5M1	2.05	0.024161131
MYB101200005680	MYB	-3.58	0.059828677
MYB101200005682	MYB	-4.57	0.043486815
MYB101200006299	MYB	-3.63	0.064399029
MYB101200006300	MYB	-3.78	0.070681108

MYB101200006301	MYB	-4.91	0.067497952
MYO5A101200004236	MYO5A	-1.7	0.027554787
N4BP2L1101200005894	N4BP2L1	2.13	0.022061768
NEDD9101200004611	NEDD9	-3.08	0.015384981
NOD1101200000816	NOD1	3.1	0.030479518
NPG1101200000931	PG2	-18.76	0.036321944
NPG4101200002138	NPG4	-15.42	0.036337771
OSBPL1A101200012925	OSBPL1A	2.73	0.020928939
OXCT1101200002213	OXCT1	-2.6	0.034839926
PAF1101200018249	PAF1	-1.48	0.034839926
PARP9101200017685	PARP9	2.07	0.027387323
PCCB101200002176	PCCB	-3.77	0.029824954
PCDHGC3101200009132	PCDHGC3	-3.74	0.012427897
PDE8B101200008833	PDE8B	-3.08	0.015384981
PDZD8101200004737	PDZD8	1.91	0.025289141
PEAR1101200006086	PEAR1	-35.49	0.013229164
PIGC101200001537	PIGC	1.75	0.030834869
PLCB2101200003045	PLCB2	-3.59	0.043486815
PLEKHA5101200011532	PLEKHA5	-8.73	0.020791775
PLEKHG5101200012602	PLEKHG5	-4.69	0.011325959
PLIN2101200002474	PLIN2	3.39	0.023625186
PPP2R5B101200007662	PPP2R5B	-2.72	0.05464693
PROC101200002193	PROC	7.31	0.051388421
PROX1101200001063	PROX1	2.21	0.058322938
PTPRO101200011537	PTPRO	4.22	0.017958814
PTPRO101200011538	PTPRO	4.49	0.017263058
QSOX1101200015402	QSOX1	-46.74	0.030479518
QSOX1101200015403	QSOX1	-3.45	0.012427897
RGS4101200001680	RGS4	5.29	0.023062814
SDC2101200004356	SDC2	-15.89	0.04251808
SELENBP1101200006173	SELENBP1	-3.35	0.024442551
SEMA4F101200010077	SEMA4F	-8.66	0.013505815
SGK3101200010692	SGK3	3.58	0.027089529
SLC24A4101200004436	SLC24A4	-4.37	0.043602104
SLC24A4101200013755	SLC24A4	-4.36	0.050988395
SLC27A3101200006143	SLC27A3	-3.29	0.042330869
SLC38A6101200006962	SLC38A6	2.81	0.013923221
SLC39A14101200003629	SLC39A14	-5.41	0.055608279
SLC6A12101200011658	SLC6A12	-3.91	0.034472398
SLC6A12101200011659	SLC6A12	-3.4	0.020362309
SLC9A1101200000140	SLC9A1	-4.12	0.026266659
SPATS2L101200001605	SPATS2L	-4.23	0.043482908
SPOCK2101200003741	TESTICAN	-2.33	0.015384981
SSFA2101200018575	ZSWIM2	5.94	0.02382939
TCF7L2101200005517	TCF7L2	-2.98	0.077643723
TCF7L2101200018233	TCF7L2	-3.18	0.058265406
TEX2101200016336	TEX2	-2.14	0.024686261
TGFBR3101200002546	TGFBR3	-31.22	0.034331328
TLR1101200000222	TLR1	3.44	0.020517657
TLR4101200000755	TLR4	6.33	0.075473394
TLR5101200000982	TLR5	6.44	0.026317431
TMEM70101200004706	TMEM70	2.82	0.034839926
TNF101200002297	TNFA	-5.33	0.02716987

TPM1101200000541	TPM1	-4.67	0.016106629
TRERF1101200005170	TRERF1	-2.91	0.034331328
TSPAN6101200002907	TSPAN6	10.83	0.022307086
TTF2101200005964	TTF2	-1.77	0.027699756
TTLL7101200012973	TTLL7	-7.79	0.045131281
TYRO3101200006695	TYRO3	-4.66	0.02966989
UHRF1101200008187	UHRF1	-3.32	0.042330869
USP32101200016753	USP32	-1.48	0.028624423
VCL101200002209	VCL	-2.74	0.020517657
VSIG4101200020179	VSIG4	6.39	0.023625186
XCR1101200000032	XCR1	-29.81	0.025306219
ZBTB48101200012612	ZBTB48	-2.08	0.018624456
ZC3H12C101200014886	ZC3H12C	-2.47	0.037011112
ZEB1101200015769	ZEB1	-2.01	0.032518445
ZNF496101200008680	ZNF496	-4.28	0.04550979

*Positive fold change values indicate up-regulation of gene expression in $CD1^+$ vs. $CD1^-$ cDC populations, whereas, negative fold change values indicate up-regulation of gene expression in $CD1^-$ vs. $CD1^+$ cDC populations.

Supplementary Table S2. Differential TLR gene expression in porcine blood $CD1^+$ and $CD1^-$ cDC populations

**TLR**	**Fold difference**	**Adjusted P value**
TLR1	3.44	0.020517657
TLR2	2.04	0.023381299
TLR3	-1.91	0.168498089
TLR4	6.33	0.075473394
TLR5	6.44	0.026317431
TLR7	-1.11	0.765962298
TLR8	-1.16	0.739426372
TLR9	-1.78	0.315949405

*Positive fold change values indicate up-regulation of gene expression in $CD1^+$ vs. $CD1^-$ cDC populations, whereas, negative fold change values indicate up-regulation of gene expression in $CD1^-$ vs.$CD1^+$ cDC populations.

Supplementary Table S3. Sources and public access for the human and murine gene expression datasets analysed in this paper.

Platform	GEO Series Accession number	Cell Population[a]	GEO Sample Accession number
Illumina HumanHT -12 V4.0 expression bead chip	GSE35459	Blood $CD1c^{+}$ cDC (5)	GSM868904<br>GSM868905<br>GSM868906<br>GSM868907<br>GSM868908
Illumina HumanHT -12 V4.0 expression bead chip	GSE35459	Blood $CD14^{+}$ $CD16^{+}$ Monocyte (6)	GSM868883<br>GSM868884<br>GSM868885<br>GSM868886<br>GSM868887<br>GSM868888
Illumina HumanHT -12 V4.0 expression bead chip	GSE35459	Blood $CD141^{+}$ cDC (5)	GSM868894<br>GSM868895<br>GSM868896<br>GSM868897<br>GSM868898
Affymetrix Mouse Gene 1.0 ST Array	GSE15907	Spleen $CD8^{-}4^{-}11b^{+}$ cDC (3)	GSM538265<br>GSM538266<br>GSM538267
Affymetrix Mouse Gene 1.0 ST Array	GSE15907	Spleen CD4+ dendritic cell (DC.4+.Sp) (5)	GSM538248<br>GSM538249<br>GSM538250<br>GSM605826<br>GSM538251
Affymetrix Mouse Gene 1.0 ST Array	GSE15907	Blood conventional monocyte (Mo.6C+II-.Bl) (3)	GSM605872<br>GSM605873<br>GSM605874
Affymetrix Mouse Gene 1.0 ST Array	GSE15907	Spleen $CD8\alpha^{+}$ cDC (DC.8+Sp.ST) (5)	GSM538258<br>GSM538259<br>GSM538260<br>GSM538261<br>GSM605827

[a] The number of replicates is shown in parentheses.

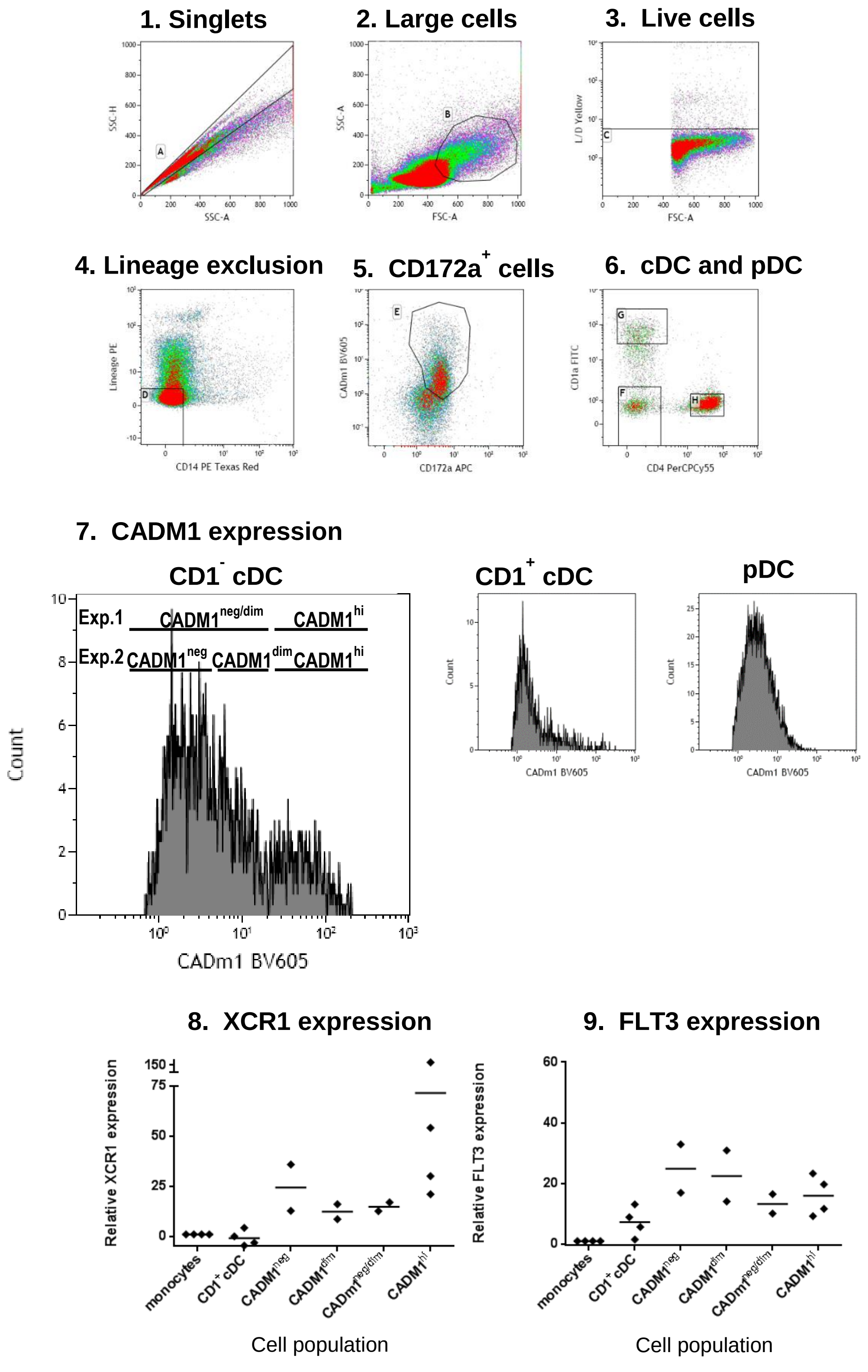

Supplementary Figure S5: Assessment of CADM1, XCR1 and FLT3 expression on porcine blood DC. Enriched DCs were stained with mAbs and analysed by flow cytometry. Illustrative dot plots show the gating strategy: (**1**) singlet, (**2**) large mononuclear (**3**) live, (**4**) CD14^{-} and lineage marker^{-} (CD3, CD8α, CD21); (**5**) CD172a^{+} and CADM1^{+} DC (**6**) CD1^{-} cDC, CD1^{+-} cDC, and CD4^{+} pDC, and (**7**) assessment of CADM1 expression on each population. (**8**) XCR1 and FLT3 expression on sorted cell populations was determined by RT-qPCR and shown relative to monocytes. CD1^{-} cDC were delineated into CD1^{-}CADM1$^{neg/dim}$ and CD1^{-}CADM1^{hi} populations in the first instance (Exp1) and CD1^{-}CADM1^{neg}, CD1^{-}CADM1^{dim} and CD1^{-}CADM1^{hi} populations in the second experiment (Exp 2) (**7**).

Supplementary Materials and Methods

XCR1 and FLT3 RT-qPCR analysis of sorted cDC populations

In order to assess XCR1 and FLT3 gene expression associated with cDC populations RT-qPCR assays were performed on sorted populations. $CD1^{-}CADM1^{neg}$, $CD1^{-}CADM1^{dim}$ and $CD1^{-}CADM1^{hi}$ populations were sorted following staining with antibodies as described in the materials and methods with the addition of biotinylated anti-CADM1 (clone 3E1, MBL, Caltag Medsystems, Buckingham, UK), followed by Streptavidin-Brilliant Violet 605 (Biolegend, London, UK).

Total RNA from sorted DCs (and monocytes as the reference population) was extracted using the RNeasy Micro Kit (Qiagen, Manchester, UK) according to the manufacturer's instructions. Genomic DNA present was removed using RNase-Free DNase Set (Qiagen). RNA was reverse transcribed using random hexamers and the M-MLV Reverse Transcriptase (Promega, Southampton, UK). The cDNA obtained for each population was evaluated for expression of XCR1 or FLT 3 by RT-qPCR. All qPCR reactions were performed using SYBR® Select Master Mix (Thermo Fisher Scientific, Paisley, UK) in a final volume of 20µl. The primers used were XCR1 (Forward, 5′-CGATGCCGTCTTCCACAAG-3′, and Reverse, 5′-GGAACCACTGGCGTTCTGA-3′, Marquet et al 2014), FLT3 (Forward, 5′-TGTTCACGCTGAATATAAGAAGGAA-3′ and Reverse, 5′-GGAGCAGGAAGCCTGACTTG-3′, Marquet et al., 2014) and BACT (Forward, 5'-GACTCAGATCATGTTCGAGACCTT-3', and Reverse, 5'-CATGACAATGCCAGTGGTGC-3'). Analysis was performed using the MxPro QPCR Software (Agilent Technologies, Stockport, UK) and the cycle threshold (C_T) values for each amplification curve were determined. Relative quantification was calculated using the 2-ΔCt method (Real time PCR, Bio-Rad) and normalized to expression of the reference gene BACT (β-actin) using monocytes as a calibrator sample.
